# *PD*-*L1* and *PD*-*L2* expression correlated genes in non-small-cell lung cancer

**DOI:** 10.1186/s40880-019-0376-6

**Published:** 2019-06-03

**Authors:** Trine Vilsbøll Larsen, Dianna Hussmann, Anders Lade Nielsen

**Affiliations:** Department of Biomedicine, Aarhus University, 8000 Aarhus C, Denmark

**Keywords:** Non-small-cell lung cancer, Biomarker, Immunotherapy, Immune checkpoints, Interferon, Chr9p24, PD-L1, PD-1, PD-L2

## Abstract

**Background:**

Programmed cell death ligand-1 (PD-L1) and ligand-2 (PD-L2) interaction with programmed cell death protein-1 (PD-1) represent an immune-inhibiting checkpoint mediating immune evasion and is, accordingly, an important target for blockade-based immunotherapy in cancer. In non-small-cell lung cancer (NSCLC), improved understanding of PD-1 checkpoint blockade-responsive biology and identification of biomarkers for prediction of a clinical response to immunotherapy is warranted. Thus, in the present study, we systematically described *PD*-*L1* and *PD*-*L2* expression correlated genes in NSCLC.

**Methods:**

We performed comparative retrospective analyses to identify *PD*-*L1* and *PD*-*L2* mRNA expression correlated genes in NSCLC. For this, we examined available datasets from the cancer cell line encyclopedia (CCLE) project lung non-small-cell (Lung_NSC) and the cancer genome atlas (TCGA) projects lung adenocarcinoma (LUAD) and squamous cell carcinoma (LUSC).

**Results:**

Analysis of the CCLE dataset Lung_NSC identified expression correlation between *PD*-*L1* and *PD*-*L2*. Moreover, we identified expression correlation between 489 genes and *PD*-*L1*, 191 genes and *PD*-*L2*, and 111 genes for both. *PD*-*L1* and *PD*-*L2* also expression correlated in TCGA datasets LUAD and LUSC. In LUAD, we identified expression correlation between 257 genes and *PD*-*L1*, 914 genes and *PD*-*L2*, and 211 genes for both. In LUSC, we identified expression correlation between 26 genes and *PD*-*L1*, 326 genes and *PD*-*L2*, and 13 genes for both. Only a few genes expression correlated with *PD*-*L1* and *PD*-*L2* across the CCLE and TCGA datasets. Expression of Interferon signaling-involved genes converged in particular with the expression correlated genes for *PD*-*L1* in Lung_NSC, for *PD*-*L2* in LUSC, and for both *PD*-*L1* and *PD*-*L2* in LUAD. In LUSC, *PD*-*L1*, and to a lesser extent *PD*-*L2,* expression correlated with chromosome 9p24 localized genes, indicating a chromosome 9p24 topologically associated domain as an important driver of in particular LUSC *PD*-*L1* expression. Expression correlation analyses of the PD-L1 and PD-L2 receptors programmed cell death protein-1 (PD-1), Cluster of differentiation 80 (CD80), and Repulsive guidance molecule B (RGMB) showed that *PD*-*1* and *CD80* expression correlated with both *PD*-*L1* and *PD*-*L2* in LUAD. *CD80* expression correlated with *PD*-*L2* in LUSC.

**Conclusions:**

We present gene signatures associated with *PD*-*L1* and *PD*-*L2* mRNA expression in NSCLC which could possess importance in relation to understand PD-1 checkpoint blockade-responsive biology and development of gene signature based biomarkers for predicting clinical responses to immunotherapy.

**Electronic supplementary material:**

The online version of this article (10.1186/s40880-019-0376-6) contains supplementary material, which is available to authorized users.

## Background

**Fig. 1:**
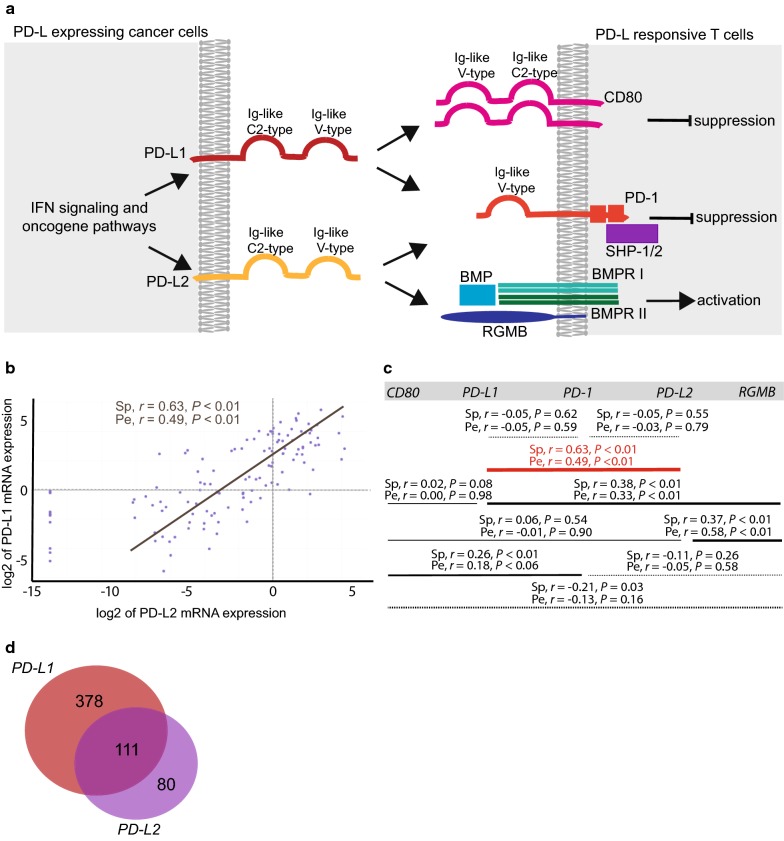
*PD*-*L1* and *PD*-*L2* expression correlation analyses in 114 NSCLC cell lines from the CCLE dataset (Lung_NSC). **a** Overview of key factors interacting with PD-L1 and PD-L2 in NSCLC. PD-L1 and PD-L2 are ligands of PD-1 and the interaction of PD-L1 or PD-L2 with PD-1 results in T cell suppression. Moreover, PD-L1 can interact with CD80 on activated T cells and inhibit T cell activity. PD-L2 has a second receptor, RGMB, and this interaction can activate T cells. **b** Expression correlation analysis of *PD*-*L1* and *PD*-*L2* in the CCLE dataset (Lung_NSC, n = 114) using Broad portal analysis of RNA sequencing-based gene expression data. Spearman and Pearson correlation coefficients *r* and the corresponding *P* value are shown. **c** Gene expression correlation between *PD*-*1*, *CD80*, *RGMB, PD*-*L1*, and *PD*-*L2* in CCLE dataset (Lung_NSC, n = 114) Spearman and Pearson correlation coefficients *r* and the corresponding *P* values are shown. The horizontal lines show the pairwise comparison of genes and line thickness is proportional to the degree of expression correlation. Dashed lines indicate negative expression correlation. Significant expression correlation is indicated in red. **d** Number of genes expression correlated with *PD*-*L1*, *PD*-*L2*, and both in CCLE dataset (Lung_NSC, n = 114). For all panels, the criteria for significant expression correlation were Pearson correlation coefficient *r* ≥ 0.3 or ≤ − 0.3, Spearman correlation coefficient *r* ≥ 0.4 or ≤ − 0.4, and *P* values < 0.05. The analysis was performed using the GenomicScape portal. *BMP* bone morphogenic protein, *BMPR I* BMP receptor type 1, *BMPR II* BMP receptor type 2, *C2-type* constant 2-type, *CD80* cluster of differentiation 80, *IFN* interferon, *Ig-like* Immunoglobulin-like, *PD-1* programmed cell death protein-1, *PD-L1* programmed cell death ligand-1, *PD-L2* programmed cell death ligand-2, *RGMB* repulsive guidance molecule B, *SHP-1/2* Src homology 2 domain-containing protein tyrosine phosphatase 1 and 2, *Pe* Pearson, *r* correlation coefficient, *Sp* Spearman, *V-type* variable-type

Non-small-cell lung cancer (NSCLC), the most common form of lung cancer, is a heterogeneous disease with adenocarcinoma and squamous cell carcinoma being the predominant molecular subtypes [1]. Antibody-mediated blockade of programmed cell death protein-1 (PD-1) or cell-surface localized programmed cell death ligand-1 (PD-L1) provides a novel therapeutic paradigm for patients with advanced NSCLC [2–6]. However, only a subset of the NSCLC patients benefits from immunotherapy with PD-L1/PD-1 axis blockade [2, 6]. PD-1 is inducibly expressed on cluster of differentiation 4 positive (CD4+) T cells, cluster of differentiation 8 positive (CD8+) T cells, regulatory T cells, natural killer T cells, B cells, and activated monocytes [7–11]. PD-1 expression is induced by T cell receptor and B cell receptor signaling and augmented by tumor necrosis factor (TNF) stimulation [7–9]. Besides binding to PD-L1, PD-1 can bind to the cell-surface of localized programmed cell death ligand-2 (PD-L2). PD-L1 and PD-L2 compete for binding to PD-1. The interaction of PD-L2/PD-1 shows a 6-fold higher affinity compared to the interaction of PD-L1/PD-1, but the generally lower expression level of PD-L2 may favor PD-L1 as the primary ligand for PD-1 [12]. The binding of PD-1 ligands to PD-1 prevent a cytotoxic T cell response against the tumor cells by inhibiting kinases involved in T cell activation [9, 13–15]. Whereas PD-L1/PD-1 interaction blocks the activation of most T cell subtypes, the PD-L2/PD-1 interaction has been proposed to primarily inhibit the CD4+ T helper 2 subsets (Th2) response [16]. Additionally, PD-L1, but not PD-L2, can bind to the cluster of differentiation 80 (CD80), which can result in a bidirectional immune inhibitory response [17]. Conversely, PD-L2, but not PD-L1, can bind the repulsive guidance molecule B (RGMB) receptor, a co-receptor for bone morphogenetic protein (BMP) [18]. The PD-L2/RGMB interaction co-stimulates CD4+ T cell responses and promotes T helper 1 subset (Th1) polarization [19]. The molecular interactions involving PD-L1 and PD-L2 are illustrated schematically in Fig. 1a. The *PD*-*L1* and *PD*-*L2* genes are tandem localized at Chr9p24.1 with a distance of 60 kb between the cognate promoters. PD-L1 is expressed on a variety of immune cells and cancer cells, including NSCLC cells [3, 11, 15]. PD-L2 is expressed on macrophages, dendritic cells and cancer cells, including NSCLC cells [3, 11]. In general, PD-L2 expression is less prevalent than PD-L1 expression in cancer cells [16]. In the tumor microenvironment, *PD*-*L1* and *PD*-*L2* mRNA expression can be induced by immune cell-secreted inflammatory cytokines, such as Interferon-alpha (IFN-α), Interferon-beta (IFN-β), Interferon-gamma (IFN-γ), Interleukin 4, Interleukin 10, and granulocyte/macrophage colony-stimulating factor, resulting in adaptive immune resistance [15, 16, 20, 21]. *PD*-*L1* and *PD*-*L2* mRNA expression can also be upregulated due to cancer cell-autonomous mechanisms, for example, mutation depending oncogenic signaling, resulting in intrinsic immune resistance [22].

Tumor PD-L1 expression in NSCLC has been shown to be associated with poor prognosis in two meta-analyses [23, 24]. Moreover, meta-analyses of clinical trials have illustrated an overall positive response to immunotherapy with PD-L1/PD-1 axis blockade in NSCLC patients and positive correlation between the PD-L1 tumor proportion score (TPS) (the percentage of PD-L1 expressing tumor cells determined by immunohistochemistry) and the response rate [25, 26]. In the CheckMate 017 and CheckMate 057 trials corresponding to squamous and non-squamous NSCLC patients, respectively, patients receiving the PD-1 antibody, nivolumab, as a second-line of treatment showed a response rate of 20% regardless of PD-L1 TPS [4, 5]. Analysis of subgroups defined by PD-L1 expression showed that PD-L1 TPS ≥ 1%, ≥ 5%, and ≥ 10% were associated with improved response rates (31%, 36%, and 37%, respectively) in the CheckMate 057, whereas, an association between PD-L1 TPS and response rate was not evident from the CheckMate 017 [4, 5]. In the KEYNOTE-001 and KEYNOTE-24 trials, which were the first trials to use first- and second-line therapies with PD-1 antibody, pembrolizumab, in advanced NSCLC patients, PD-L1 TPS ≥ 50% resulted in response rates of 52% and 45%, respectively [27–29]. The PD-L1 antibodies, atezolizumab, durvalumab, and avelumab, also showed promising response rates as immunotherapy in NSCLC even with low PD-L1 TPS [6, 30]. Unlike the PD-1 antibodies, the PD-L1 antibodies also block the PD-L1/CD80 axis but leave the PD-L2/PD-1 axis unaffected. The response rate in NSCLC was similar for PD-1 and PD-L1 antibody immunotherapy but less immune-related adverse effects were observed from the latter [31]. The efficient stratification of immunotherapy responders and non-responders among NSCLC patients remains a major challenge [2, 3, 6, 32]. Given the potential overlapping involvement of PD-L1 and PD-L2 for defining immune checkpoint inhibition, we performed this retrospective expression correlation study to identify genes in NSCLC having expression profiles closely associated with the *PD*-*L1* and *PD*-*L2* expression profiles. The presented study illustrates novel aspects of *PD*-*L1* and *PD*-*L2* regulation, with potential biological relevance, as well as relevance for immunotherapy response stratification.

## Methods and materials

### Datasets

The following datasets were used for retrospective gene expression analyses. The cancer cell line encyclopedia (CCLE) project dataset is a compilation of gene expression data from human cancer cell lines [33]. The CCLE subset, lung_non-small-cell (Lung_NSC), contains gene expression data from NSCLC cell lines, were obtained from the CCLE portal (https://portals.broadinstitute.org/ccle/home). The Cancer Genome Atlas (TCGA) project dataset is a comprehensive atlas of gene expression and gene regulation across human cancers [34]. The TCGA data subsets, lung adenocarcinoma (LUAD) and lung squamous cell carcinoma (LUSC), contain gene expression data from dissected NSCLC tumors, were obtained from the TCGA data portal (https://tcga-data.nci.nih.gov). The Genotype-Tissue Expression (GTEx) project dataset is a comprehensive atlas database of gene expression in human tissues [35]. The GTEx lung subset, contains RNA sequencing-based expression data from normal lung tissue, were obtained from the GTEx portal (http://www.gtexportal.org/home/).

### Broad portal analysis

The Broad portal (https://portals.broadinstitute.org/ccle) was used to generate scatter plots from CCLE Lung_NSC expression data for *PD*-*L1* and *PD*-*L2* using Illumina RNA sequencing data (processed with normalization followed by Log2 transformation).

### GenomicScape portal analysis

The portal GenomicScape (http://www.genomicscape.com/) was used to analyze CCLE Lung_NSC expression data based on deposited microarray data from a robust multi-array average (RMA) normalized Affymetrix Human Genome U133 Plus 2.0 Arrays. The GenomicScape co-expression module was used to calculate pairwise expression correlations between *PD*-*L1*, *PD*-*L2*, *IFNG*, *IFNGR1*, *IFNGR2*, *STAT1*, *JAK1*, *JAK2*, *IRF1*, *IRF9*, *TYK2*, *STAT2*, *STAT3*, *IFNAR1*, *IFNAR2*, *PD*-*1*, *CD80*, and *RGMB*, as well as to generate gene lists composed of expression correlated genes for *PD*-*L1*, *PD*-*L2*, *IRF1* and *IRF9*.

### Wanderer portal analysis

The Maplab tool Wanderer (http://maplab.imppc.org/wanderer/) was used to calculate the mRNA expression levels of *PD*-*L1*, *PD*-*L2*, *PD*-*1*, *CD80*, and *RGMB* with corresponding Wilcoxon *P* values in GTEx and TCGA datasets using RNA sequencing data (in format Log2(normalized_RSEM+1)).

### cBioPortal analysis

TCGA RNA sequencing data (in format V2 RSEM Z-score) were in the cBioPortal for Cancer Genomics interface (http://www.cbioportal.org/) used to generate gene lists composed of expression correlated genes for *PD*-*L1*, *PD*-*L2*, *IRF1*, and *IRF9*. Correlation coefficients were calculated before the Log2 transformation of RNA sequencing data. Log2 transformed TCGA RNA sequencing data were used for heat map analyses and hierarchical clustering. Finally, the plot function in the cBioPortal interface was used to calculate correlation coefficients between PD-L1 protein expression (from reverse-phase protein arrays) and mRNA expression of identified *PD*-*L1* and *PD*-*L2* expression correlated genes.

### Xena portal analysis

UCSC Xena browser (https://xenabrowser.net/) was used to calculate pairwise expression correlation using the GTEx lung and TCGA RNA sequencing data (in format Log2[normalized_counts+1]). Expression correlation coefficients and corresponding *P* values were calculated before Log2 transformation using the browser http://gepia.cancer-pku.cn/ which analyses data present in the Xena portal.

### Gene set enrichment analysis (GSEA)

The molecular signatures database (MSigDB) is a collection of annotated gene sets [36, 37]. GSEA was used to compute overlaps between gene sets representing expression correlated genes for *PD*-*L1*, *PD*-*L2*, Interferon response factor 1 (*IRF1*), and Interferon response factor 9 (*IRF9*) and available gene sets in MSigDB (http://software.broadinstitute.org/gsea/). We analyzed the MSigDB gene set collection named hallmark gene sets, which summarized and represented specific well-defined biological states or processes [36, 37]. Moreover, we analyzed the MSigDB gene set collection named positional gene sets, corresponding to each human chromosome and each cytogenetic band that contained at least one gene. Values in the tables presenting GSEA represented the number of genes in the respective MSigDB hallmark gene sets (K), the number of genes in the examined *PD*-*L1*, *PD*-*L2*, *IRF1*, and *IRF9* expression correlation gene signatures overlapping with the MSigDB hallmark gene set (k), *P* value representing the hypergeometric distribution of overlapping genes, and the false discovery rate (FDR) *q* value after correction for multiple hypothesis testing. Enriched gene signatures with *q* value < 1E−03 were considered significant. The list of the specific gene sets analyzed and their sources are available in supplementary tables.

### Statistical analysis

To decrease data overfitting, the criteria used for significant expression correlation between two genes was a Pearson correlation coefficient *r* ≥ 0.3 or ≤ − 0.3, a Spearman correlation coefficient *r* ≥ 0.4 or ≤ − 0.4, and all corresponding *P* values < 0.05. Notably, cBioPortal and Xena portal analyses included gene expression data for different numbers of TCGA LUAD and LUSC patients resulting in minor differences in obtained correlation coefficients and resulting statistics.

## Results

### Analysis of *PD*-*L1* and *PD*-*L2* expression correlated genes in CCLE dataset

We first addressed *PD*-*L1* and *PD*-*L2* mRNA expression in 114 NSCLC cell lines from the CCLE project Lung_NSC. Retrospective data analyses showed that *PD*-*L1* and *PD*-*L2* mRNA expression was correlated (Fig. 1b, c). *PD*-*L2* mRNA expression was in general 2- to 4-fold lower than *PD*-*L1* mRNA expression (Fig. 1b). Neither *PD*-*L1* nor *PD*-*L2* expression correlated with their respective receptors PD-1, CD80, and RGMB with the criteria for significant expression correlation being a Spearman correlation coefficient *r* ≥ 0.4 or ≤ − 0.4, a Pearson correlation coefficient *r* ≥ 0.3 or ≤ − 0.3, and all *P* values < 0.05 (Fig. 1c). Notably, correlation between *RGMB* and *PD*-*L1* expression, as well as between *RGMB* and *PD*-*L2* expression, was near significance (Fig. 1c). We next identified *PD*-*L1* and *PD*-*L2* expression correlated genes in the CCLE dataset Lung_NSC. 489 genes expression correlated with *PD*-*L1*, 191 genes expression correlated with *PD*-*L2*, and 111 genes expression correlated with both *PD*-*L1* and *PD*-*L2* (Fig. 1d and Additional file 1: Table S1). Interestingly, GSEA revealed that *PD*-*L1* and *PD*-*L2* expression correlated genes were enriched to the hallmark gene sets TNFα signaling via Nuclear factor kappa B (NFKB) (genes regulated by NF-kB in response to TNF), Kirsten rat sarcoma viral oncogene homolog (KRAS) signaling (genes up-regulated by KRAS activation), and Transforming growth factor β (TGFβ) signaling (genes up-regulated in response to TGFB1) (Additional file 2: Fig. S1B and Additional file 3: Table S2). GSEA also showed that *PD*-*L1* and *PD*-*L2* expression correlated genes were enriched for belonging to the epithelial-mesenchymal-transition (EMT) hallmark gene set (genes defining the epithelial-mesenchymal-transition, as in wound healing, fibrosis, and metastasis) (Additional file 2: Fig. S1B and Additional file 3: Table S2). We note a previously described association between EMT, PD-L1 expression, and immunosuppression [38, 39]. GSEA showed that among the hallmark gene sets enriched with *PD*-*L1* expression correlated genes, but not with *PD*-*L2* expression correlated genes, were the hallmark gene sets IFN-α response (genes up-regulated in response to alpha interferon proteins) and IFN-γ response (genes up-regulated in response to IFNγ) (Additional file 3: Table S2). We next addressed how individual genes involved in IFN signaling expression correlated with *PD*-*L1* and *PD*-*L2* in Lung_NSC (Additional file 4: Table S3). Genes in the canonical signaling pathway for IFN-γ include IFN-γ receptor 1 and 2 (*IFNGR1 and IFNGR2*), Janus kinase 1 and 2 (*JAK1 and JAK2*), Signal transducer and activator of transcription 1, 2, and 3 (*STAT1*, *STAT2*, *and STAT3*), and Interferon regulatory factor 1 (*IRF1*). Genes in the canonical signaling pathway for IFN-α include IFN-α receptor 1 and 2 (*IFNAR1* and *IFNAR2*), *JAK1* and Tyrosine kinase 2 (*TYK2*), *STAT1*, *STAT2*, and *STAT3*, and Interferon regulatory factor 9 (*IRF9*) [21]. In Lung_NSC, 152 genes expression correlated with *IRF1* and 248 genes expression correlated with *IRF9* (Additional file 2: Fig. S1A and Additional file 5: Table S4). Interestingly, *IRF9*, but not *IRF1*, expression correlated with *PD*-*L1* (Additional file 4: Table S3). *IRF1* and *IRF9* expression correlated not with *PD*-*L2* (Additional file 4: Table S3). We also examined whether the identified Lung_NSC *PD*-*L1* and *PD*-*L2* expression correlated genes (Additional file 1: Table S1) were represented among the identified Lung-NSC *IRF1* and *IRF9* expression correlated genes (Additional file 5: Table S4). Of the *PD*-*L1* expression correlated genes, 10% (49/489) expression correlated with *IRF1*, and 17% (83/489) expression correlated with *IRF9* (Additional file 2: Fig. S1A). Of the *PD*-*L2* expression correlated genes, 6% (11/191) expression correlated with *IRF1*, and 5% (9/191) expression correlated with *IRF9* (Additional file 2: Fig. S1A). *IRF1* and *IRF9* were not expression correlated with *PD*-*1*, *CD80,* and *RGMB* (Additional file 4: Table S3). GSEA showed extensive overlaps between hallmark gene sets enriched with *PD*-*L1* and *PD*-*L2* expression correlated genes and hallmark gene sets enriched with *IRF1* and *IRF9* expression correlated genes (Additional file 2: Fig. S1B, Additional file 3: Table S2, and Additional file 6: Table S5).

### *PD*-*L1* and *PD*-*L2* expression correlated genes are differently associated with IFN signaling and immune cell markers in NSCLC tumor samples

**Fig. 2:**
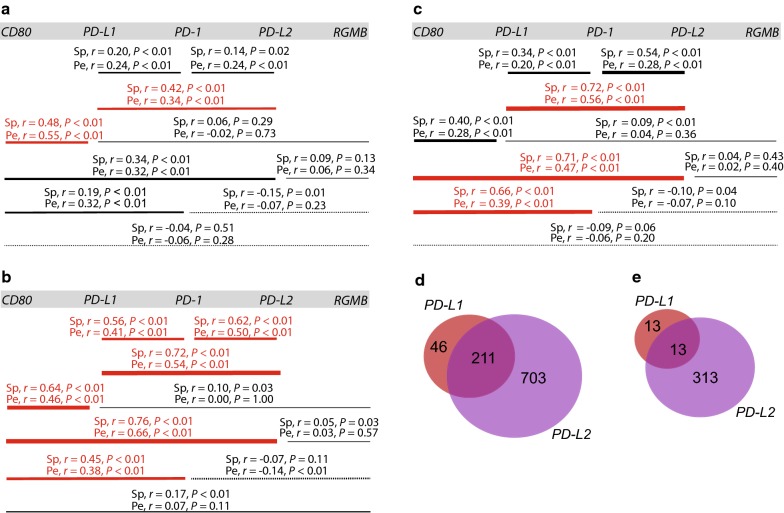
mRNA expression correlation analysis of *PD*-*L1* and *PD*-*L2* in GTEx lung dataset and TCGA datasets (LUAD and LUSC) reveals major differences in mRNA expression profiles. **a**–**c** Expression correlation between *PD*-*L1*, *PD*-*L2* and mRNA for cognate receptors CD80, PD-1, and RGMB in normal lung tissue from GTEx lung (n = 387) **a**, TCGA dataset LUAD (n = 706) **b**, and TCGA dataset LUSC (n = 626) **c** using Xena portal data. Spearman and Pearson correlation coefficients *r* and their corresponding *P* values are shown. The horizontal lines show the pairwise comparison of genes and line thickness is proportional to the degree of expression correlation. Dashed lines indicate negative expression correlation. Significant expression correlation is indicated in red. **d**, **e** Venn diagrams illustrating the number of genes expression correlated with *PD*-*L1, PD*-*L2*, and both, in TCGA dataset LUAD (n = 517) **d**, and LUSC (n = 501) **e**, using cBioPortal. For all panels, the criteria for significant expression correlation were Pearson correlation coefficient *r* ≥ 0.3 or ≤ − 0.3, Spearman correlation coefficient *r* ≥ 0.4 or ≤ − 0.4, and *P* values < 0.05. *Pe* Pearson, *r* correlation coefficient, *Sp* Spearman

To investigate *PD*-*L1* and *PD*-*L2* mRNA expression in NSCLC tumor samples we analyzed available RNA sequencing data from the TCGA projects LUAD and LUSC datasets, as well as RNA sequencing data for normal lung tissue from the GTEx project. In the LUAD and LUSC datasets, *PD*-*L1* expression was 2- to 3-fold higher than *PD*-*L2* expression (Additional file 7: Table S6). *PD*-*L1* and *PD*-*L2* expression correlated in LUSC and LUAD tumor samples, and as well as in normal lung tissue (Fig. 2a–c). In LUAD, both *PD*-*1* and *CD80* expression correlated with *PD*-*L1*, as well as with *PD*-*L2* (Fig. 2b). In LUSC, *CD80* expression correlated with *PD*-*L2* (Fig. 2c). In the LUAD and LUSC datasets, no expression correlation between *RGMB* and *PD*-*L1* or between *RGMB* and *PD*-*L2* was identified (Fig. 2b, c). We note that *CD80* expression correlated with *PD*-*1* in both the LUAD and LUSC datasets (Fig. 2b, c).

Next, we identified *PD*-*L1* and *PD*-*L2* expression correlated genes in LUAD and LUSC datasets. For the LUAD dataset, 257 genes and 914 genes expression correlated with *PD*-*L1* and *PD*-*L2*, respectively (Fig. 2d and Additional file 1: Table S1). We noted that 211 genes expression correlated with both *PD*-*L1* and *PD*-*L2*, representing 82% of the *PD*-*L1* expression correlated genes and 23% of the *PD*-*L2* expression correlated genes (Fig. 2d). GSEA showed that *PD*-*L1* and *PD*-*L2* expression correlated genes were enriched for belonging to the same hallmark gene sets, except for the EMT gene set only being significant among the *PD*-*L2* expression correlated genes (Additional file 3: Table S2 and Additional file 8: Fig. S2B). Notably, hallmark gene sets IFN-α response and IFN-γ response were among the hallmark gene sets for both *PD*-*L1* and *PD*-*L2* expression correlated genes (Additional file 3: Table S2 and Additional file 8: Fig. S2B). In the LUSC dataset, 26 genes expression correlated with *PD*-*L1*, and 326 genes expression correlated with *PD*-*L2* (Fig. 2e and Additional file 1: Table S1). Of these, 13 genes expression correlated with both *PD*-*L1* and *PD*-*L2*, representing 50% of the *PD*-*L1* expression correlated genes but only 4% of the *PD*-*L2* expression correlated genes (Fig. 2e). GSEA showed that all hallmark gene sets enriched for *PD*-*L1* expression correlated genes also were enriched for *PD*-*L2* expression correlated genes (Additional file 3: Table S2 and Additional file 8: Fig. S2D).

We also investigated the connection between *PD*-*L1* and *PD*-*L2* expression correlated genes and IFN signaling, with IFN signaling being represented by the 13 genes *IFNG* (encoding IFN-γ), *IFNGR1*, *IFNGR2*, *IFNAR1*, *IFNAR2*, *JAK1*, *JAK2*, *TYK2*, *STAT1*, *STAT2*, *STAT3*, *IRF1*, and *IRF9* (Additional file 9: Table S7). We performed an expression correlation analysis between these 13 genes and *PD*-*L1* and *PD*-*L2* (Additional file 4: Table S3). In the LUAD dataset, *PD*-*L1* and *PD*-*L2* expression correlated with *IRF1*, *IFNG*, *STAT1*, and *JAK2* (Additional file 4: Table S3). In addition, *PD*-*L2* expression correlated with *IFNGR1* (Additional file 4: Table S3). Notably, *PD*-*L1* expression was not correlated with *IRF9*, a different scenario from that observed in CCLE Lung_NSC. In line with this, we noted that in the LUAD dataset the number of expression correlated genes with *IRF1* (559 genes) was higher than that for *IFR9* (82 genes) (Additional file 5: Table S4 and Additional file 8: Fig. S2A) which differed from the observations for CCLE Lung_NSC (Additional file 2: Fig. S1A and Additional file 5: Table S4). Prominent numbers of commonly correlated genes for *IRF1* and *PD*-*L1* (*n* = 141), as well as for *IRF1* and *PD*-*L2* (*n* = 488), were observed in LUAD (Additional file 8: Fig. S2A). This further illustrated that only 66/559 *IRF1* expression correlated genes were not *PD*-*L1* or *PD*-*L2* expression correlated (Additional file 8: Fig. S2A). In comparison, there were fewer commonly correlated genes for *IRF9* and *PD*-*L1* (*n *= 19) and for *IRF9* and *PD*-*L2* (*n* = 44) (Additional file 8: Fig. S2A). GSEA showed extensive overlapping among hallmark gene sets enriched with *PD*-*L1*, *PD*-*L2*, *IRF1,* and *IRF9* expression correlated genes (Additional file 3: Table S2, Additional file 6: Table S5 and Additional file 8: Fig. S2B).

In the LUSC dataset, of the 13 IFN signaling genes *JAK2* and *IRF1* expression correlated with *PD*-*L1* (Additional file 4: Table S3). Moreover, 10/26 of the *PD*-*L1* expression correlated genes were *IRF1* expression correlated and 3/26 of the *PD*-*L1* expression correlated genes were *IRF9* expression correlated (Additional file 8: Fig. S2C). GSEA showed that the hallmark gene sets IFN-γ response and allograft rejection (genes up-regulated during transplant rejection) were common hallmark gene sets for both *PD*-*L1* and *IRF1* expression correlated genes, as well as for *PD*-*L1* and *IRF9* expression correlated genes (Additional file 3: Table S2, Additional file 6: Table S5 and Additional file 8: Fig. S2D). In the LUSC dataset, of the 13 IFN signaling genes *IFNG*, *JAK2, STAT1*, and *IRF1* expression correlated with *PD*-*L2* (Additional file 4: Table S3). As also observed in the LUAD dataset, in the LUSC dataset the number of *IRF1* expression correlated genes (*n* = 751) was more pronounced than the number of *IFR9* expression correlated genes (*n* = 90) (Additional file 5: Table S4 and Additional file 8: Fig. S2A, C). 297/326 of the *PD*-*L2* expression correlated genes were *IRF1* expression correlated and 18/326 of the *PD*-*L2* expression correlated genes were *IRF9* expression correlated (Additional file 8: Fig. S2C). GSEA showed that *PD*-*L2*, *IRF1,* and *IRF9* expression correlated genes to a large extent belong to the same hallmark gene sets (Additional file 3: Table S2, Additional file 6: Table S5 and Additional file 8: Fig. S2D). We note that in the LUAD and LUSC datasets both *PD*-*1* and *CD80* expression correlated with *IRF1*, but not with *IRF9*, indicating the participation of IFN-γ signaling in both ligand and receptor expression during the development of immune resistance (Additional file 4: Table S3). *RGMB* expression was not correlated with *IRF1* and *IRF9* expression (Additional file 4: Table S3).

**Fig. 3:**
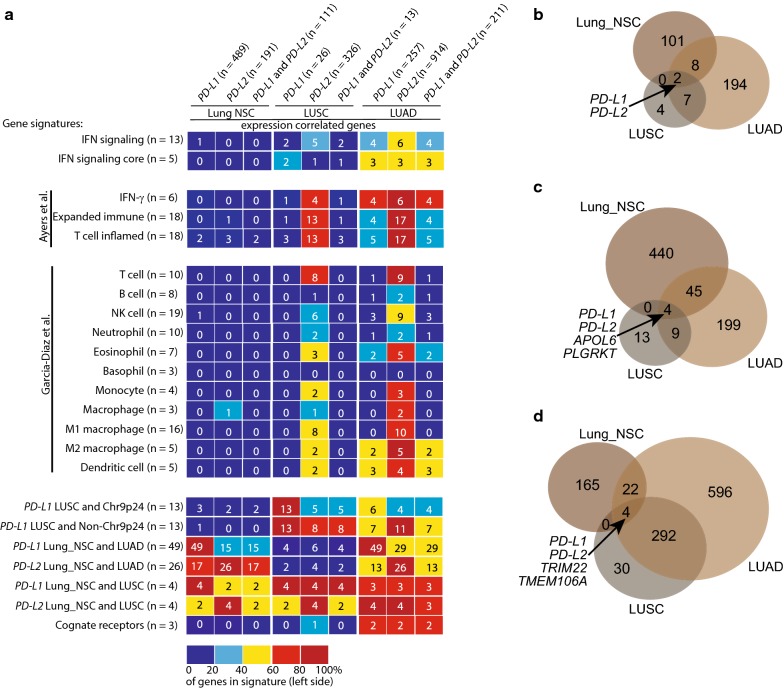
*PD*-*L1* and *PD*-*L2* expression correlated genes converge differently with immune-related gene signatures. **a** Diagram illustrating the number of common genes for various gene signatures (shown to the left) and expression correlated genes for *PD*-*L1*, *PD*-*L2*, and both, from dataset Lung_NSC, LUSC, and LUAD (shown at the top). The color indicates the percentage of the signature genes being *PD*-*L1* and *PD*-*L2* expression correlated. The gene signature IFN signaling is composed of *IFNG*, *IFNGR1*, *IFNGR2*, *IFNAR1*, *IFNAR2*, *JAK1*, *JAK2*, *TYK2*, *STAT1*, *STAT2*, *STAT3*, *IRF1*, *and IRF9*; the gene signature IFN signaling core is composed of *STAT1*, *JAK1*, *JAK2*, *IRF1*, and *IRF9*; the gene signatures IFN-γ, expanded immune, and T cell inflamed were from Ayers et al. [40]; immune cell type gene signatures are from Garcia-Diaz et al. [21]; *PD*-*L1* and *PD*-*L2* expression correlation signatures were from the present study; and the gene signature cognate receptors is composed of *PD*-*1*, *CD80,* and *RGMB*. **b**–**d** Venn diagrams illustrating the number of genes expression correlated with both *PD*-*L1* and *PD*-*L2* **b**, *PD*-*L1* **c**, and *PD*-*L2* **d** in Lung_NSC, LUAD, and LUSC. For all panels, the criteria for significant expression correlation were Pearson correlation coefficient *r* ≥ 0.3 or ≤ − 0.3, Spearman correlation coefficient *r* ≥ 0.4 or ≤ − 0.4, and *P* values < 0.05

We next addressed how *PD*-*L1* and *PD*-*L2* expression correlated genes converged with three previously described gene signatures, IFN-γ, expanded immune, and T cell inflamed, used for predicting pembrolizumab responders (Additional file 9: Table S7) [40]. The genes belonging to these gene signatures were largely included among the *PD*-*L2* expression correlated genes in the LUAD and LUSC datasets (Fig. 3a). In Lung_NSC the overlap was less evident as could be expected due to the lack of immune cells and cytokine stimulation in these in vitro grown cancer cells (Fig. 3a). The overlap between the three gene signatures and *PD*-*L1* expression correlated genes was less pronounced than that for *PD*-*L2* expression correlated genes (Fig. 3a). We also examined how previously described gene signatures representing immune cell types converged with *PD*-*L1* and *PD*-*L2* expression correlated genes in the LUAD, LUSC, and Lung_NSC datasets (Fig. 3a) (Additional file 9: Table S7) [21]. Again, the overlap between these gene signatures and *PD*-*L1* expression correlated genes was less pronounced than that for *PD*-*L2* expression correlated genes (Fig. 3a).

We analyzed the potential of *PD*-*L1* and *PD*-*L2* expression correlated genes to represent gene signatures with potential biomarker implications for immunotherapy with PD-1/PD-L1 axis blockade by searching for genes whose expression were correlated across the datasets Lung_NSC, LUAD, and LUSC. Notably, only *PD*-*L1* and *PD*-*L2* themselves remained after selecting for expression correlated genes for both *PD*-*L1* and *PD*-*L2* across all three datasets (Fig. 3b). *PD*-*L2*, plasminogen receptor with a c-terminal lysine (*PLGRKT*), and apolipoprotein L6 (*APOL6*) expression correlated with *PD*-*L1* across the Lung_NSC, LUAD and LUSC datasets (Fig. 3c and Additional file 10: Table S8). For *PD*-*L2,* the three genes *PD*-*L1*, Transmembrane protein 106A (*TMEM106A*), and Tripartite motif containing 22 (*TRIM22*) expression correlated across the Lung_NSC, LUAD, and LUSC datasets (Fig. 3d and Additional file 10: Table S8). Notably, these genes also represented the only *PD*-*L1* and *PD*-*L2* expression correlated genes across the Lung_NSC and LUSC datasets (Fig. 3c, d and Additional file 10: Table S8). The expression correlation between the identified genes, *PD*-*L1*, and *PD*-*L2* in individual LUAD and LUSC tumor samples is illustrated in Additional file 11: Fig. S3A and B. The examination of available LUSC and LUAD PD-L1 protein expression data also revealed expression correlation with PD-L1 protein except for *APOL6* mRNA (Additional file 11: Fig. S3A and B). Next, we focused on *PD*-*L1* expression correlated genes across the Lung_NSC and LUAD datasets (Additional file 10: Table S8). Here we identified 49 genes (Fig. 3c and Additional file 10: Table S8) and their expression correlation with *PD*-*L1* for individual LUAD tumors is illustrated in Additional file 11: Fig. S3C. The mRNA expression for most of these 49 genes correlated with the expression of PD-L1 protein (Additional file 11: Fig. S3C, upper panel). For *PD*-*L2*, 26 genes expression correlated across the Lung_NSC and LUAD datasets (Fig. 3d and Additional file 10: Table S8). The expression correlation of these 26 genes with *PD*-*L2* for individual LUAD tumor samples is illustrated in Additional file 11: Fig. S3C. The mRNA expression for most of these 26 genes correlated with the expression of PD-L1 protein (Additional file 11: Fig. S3C, lower panel).

### In LUSC, *PD*-*L1* expression correlated genes are enriched for being Chr9p24 localized

**Fig. 4:**
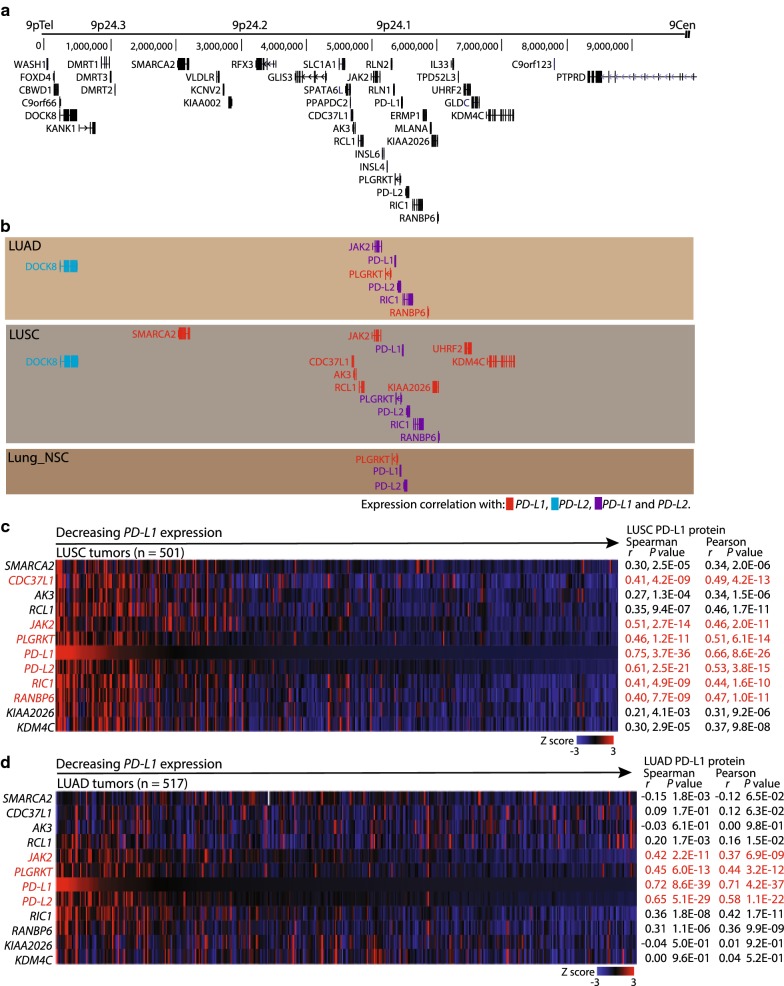
Chr9p24 localized genes display region-wide mRNA expression correlation with *PD*-*L1* in LUSC. **a** Illustration of genes located at Chr9p24 from UCSC browser. 9pTel illustrates the telomere end for the displayed region and 9Cen illustrates the direction for the localization of the Chr9 centromere region. **b** Diagram illustrating expression correlation of the genes illustrated in **a** with *PD*-*L1* and *PD*-*L2* in LUAD, LUSC, and Lung_NSC. Red color indicates expression correlation with *PD*-*L1*, the blue color indicates expression correlation with *PD*-*L2*, and purple color indicates expression correlation with both *PD*-*L1* and *PD*-*L2*. **c**, **d** Heat map analyses of mRNA expression Z-scores for *PD*-*L1* expression correlated genes located at Chr9p24 for individual patients in LUSC **c** and LUAD **d**. The heat maps are sorted relative to *PD*-*L1* mRNA expression and with the vertical gene order reflecting the position at Chr9p24. Spearman and Pearson correlation coefficients *r* and their corresponding *P* values between mRNA expression for genes of interest and PD-L1 protein expression in LUAD (n = 365) and LUSC (n = 328) are shown on the right. Correlations assigned significant are shown in red. The criteria for significant expression correlation were Pearson correlation coefficient *r* ≥ 0.3 or ≤ − 0.3, Spearman correlation coefficient *r* ≥ 0.4 or ≤ − 0.4, and *P* values < 0.05. *Pe* Pearson, *r* correlation coefficient, *Sp* Spearman

We noted fewer number of genes with *PD*-*L1* expression correlation (*n* = 26) as compared to the number of genes with *PD*-*L2* expression correlation (*n* = 326) in LUSC (Fig. 2e). GSEA for chromosomal localization of the 26 *PD*-*L1* expression correlated genes in LUSC identified enrichment for localization to the Chr9p24 region for 13/26 genes (Fig. 4a, b and Additional file 12: Table S9). Chr9p24 enrichment was also present among the *PD*-*L1* expression correlated genes in LUAD (6/257 genes), but not among the *PD*-*L1* expression correlated genes in Lung_NSC (Fig. 4b and Additional file 12: Table S9). GSEA also identified enrichment for Chr9p24 localization among the *PD*-*L2* expression correlated genes in LUSC, but for a lower fraction of genes (6/326) than observed for *PD*-*L1* (13/26) (Fig. 4b and Additional file 12: Table S9). Enrichment for localization to additional genomic regions, e.g., Chr12p13 and Chr19q13, was also present among the *PD*-*L1* and *PD*-*L2* expression correlated genes in LUAD and LUSC (Additional file 12: Table S9). In LUSC, an expression correlation between PD-L1 protein and 7/12 examinable Chr9p24-localized genes with *PD*-*L1* expression correlation was observed (note the Chr9p24-localized gene *UHRF* was excluded from the analysis) (Fig. 4c). In LUAD, the expression correlation between Chr9p24-localized genes with *PD*-*L1* expression correlation and PD-L1 protein was less pronounced (Fig. 4d). We next performed an unsorted hierarchical cluster analysis of a merged gene signature composed of genes representing the core IFN signaling, different immune cell types, and the *PD*-*L1* expression correlated genes in LUSC with Chr9p24 localization (the genes and the merged gene signature is shown in Additional file 9: Table S7). We found that in the LUSC samples the *PD*-*L1* expression correlated genes with Chr9p24 localization were preferentially clustering together (Additional file 13: Fig. S4A). Furthermore, this cluster of Chr9p24 localized genes included *PD*-*L1* and *PD*-*L2*. This point to Chr9p24 localization is the major determinant for the *PD*-*L1* and *PD*-*L2* expression profiles in LUSC (Additional file 13: Fig. S4A). The same analysis in LUAD samples showed that *PD*-*L1* and *PD*-*L2* did not cluster with the other Chr9p24-localized genes, demonstrating that in LUAD, the Chr9p24 localization was less important for determining the *PD*-*L1* and *PD*-*L2* expression profile (Additional file 13: Fig. S4B). Altogether, we conclude that the expression analyses of Chr9p24-localized genes with *PD*-*L1* expression correlation may possess relevance when searching for gene expression biomarkers that can predict responsiveness to immunotherapy with PD-1/PD-L1 axis blockade in lung squamous cell carcinoma.

## Discussion

Comparison of *PD*-*L1* and *PD*-*L2* gene expression results from the CCLE Lung_NSC cell line dataset and the TCGA LUAD and LUSC tumor datasets include some putative pitfalls. Expression analyses using the CCLE dataset identified gene expression in various cancer cell genetic backgrounds each with a unique and relative homogenous profile in terms of cancer driver and passenger mutations. However, the actual cell propagation conditions only partially mimic the context of a tumor in vivo. Gene expression analyses using the TCGA datasets (LUAD and LUSC) corresponded to incisional and core tumor biopsies, which mimic tumor cells more naturally due to stimulation of the tumor cells with factors, such as cytokines, derived from the complex tumor microenvironment. However, these biopsies may not be representative for the entire tumor burden due to tumor heterogeneity and they may as well display various degrees of tumor-infiltrating immune cells and connective tissue. With such dataset differences in mind, we still found it surprising that only *PD*-*L2*, *APOL6*, and *PLGRKT* expression were correlated with *PD*-*L1* across the CCLE Lung_NSC and TCGA (LUAD and LUSC) datasets. Similar, only the three genes *PD*-*L1*, *TRIM22*, and *TMEM106A* expression correlated with *PD*-*L2* across the three datasets. As a result, *PD*-*L2* expression should in principle be among the best indirect predictors to deduce *PD*-*L1* mRNA expression level, and vice versa. This supports the use of *PD*-*L2* expression analyses, beyond *PD*-*L1* expression analyses, to predict responsiveness to immunotherapy with PD-1/PD-L1 axis blockade in NSCLC.

Examining *PD*-*L1* and *PD*-*L2* gene expression in the NSCLC datasets separately revealed other aspects of expression regulation. In the CCLE dataset Lung_NSC, the majority of the *PD*-*L2* expression correlated genes were also expression correlated with *PD*-*L1*, 111/191 genes, but not vice versa, 111/489 genes. In the TCGA dataset LUAD, the opposite was observed, 211/914 genes and 211/257 genes, respectively. In the TCGA dataset LUSC, we observed a low number of *PD*-*L1* expression correlated genes (n = 26) relative to the number of genes expression correlated with *PD*-*L2* (n = 326). Half of these *PD*-*L1* expression correlated genes, 13/26, expression correlated with *PD*-*L2*. Thus, gene signatures representing *PD*-*L1* and *PD*-*L2* expression correlated genes seem to delineate common, as well as distinct, aspects of *PD*-*L1* and *PD*-*L2* regulation; depending on the actual NSCLC datasets analyzed.

With previous studies showing that both *PD*-*L1* and *PD*-*L2* genes are regulated by IFN signaling, we focused on IFN signaling as an important driving mechanism for the expression of *PD*-*L1* and *PD*-*L2* [21, 40]. In the CCLE dataset (Lung_NSC) the expression correlation between IFN signaling signature genes and *PD*-*L1* and *PD*-*L2* differed strikingly to the expression correlation observed in the TCGA datasets (LUAD and LUSC). *IRF9* expression correlated with *PD*-*L1* in Lung_NSC, but not in the LUAD and LUSC datasets. However, *IRF1* expression correlated with *PD*-*L1* and *PD*-*L2* expression in the LUAD and LUSC datasets indicated a shift from IFN-α involved *PD*-*L1* expression regulation in CCLE (Lung_NSC) cell lines toward IFN-γ involved *PD*-*L1* and *PD*-*L2* expression regulation in TCGA (LUAD and LUSC) tumors. *IRF1* expression correlation with *PD*-*L1* and *PD*-*L2* also goes beyond lung cancer [21]. Furthermore, our presented expression correlation data indicated a stronger correlation of *PD*-*L2* expression, relative to *PD*-*L1* expression, with active IFN signaling in LUAD and LUSC tumors.

We found it intriguing that *PD*-*L1* expression correlated with only a few other genes in the TCGA LUSC dataset, and that a prominent number of these genes were localized to Chr9p24. Of the genes located to Chr9p24 and expression correlated with *PD*-*L1*, only *PD*-*L2* and *JAK2* were associated with IFN-γ signaling. JAK2 is essential for intracellular signaling upon exposure of cancer cells to IFN-γ produced by T cells upon antigen recognition. In cancer, loss of function mutations in *JAK2* are relatively often observed and can in principle result in decreased antigen presentation, lack of T cell infiltrates, tumor cell resistance to the anti-proliferative effects of IFN-γ, and can prevent adaptive *PD*-*L1* and *PD*-*L2* expression upon IFN-γ exposure [41, 42]. One consequence could be intrinsic and/or acquired resistance to immunotherapy with PD-1/PD-L1 axis blockade [41, 42]. In LUSC, Chr9p21.3 allelic deletions of the tumor suppressor gene Cyclin-dependent kinase inhibitor 2A (*CDKN2A*) can, in addition, include co-deletion of *JAK2*, *PD*-*L1*, *PD*-*L2* [43]. Moreover, co-amplification of *JAK2* and *PD*-*L1* is observed in NSCLC and patients with *JAK2* and *PD*-*L1* co-amplification has a poorer overall survival and recurrence-free survival compared to patients with normal *PD*-*L1* copy number [44]. Copy number variation (CNV) is a characteristic of NSCLC and drives the expression profile of underlying genes [45–49]. However, we found no evidence that Chr9p CNVs should be the driving mechanism for the distinct *PD*-*L1* versus *PD*-*L2* expression correlation profile in LUSC, as such CNVs should affect *PD*-*L1* and *PD*-*L2* expression simultaneous. The architecture of the human genome is a major determinant for controlling gene expression [50, 51]. First, local enhancer-promoter interaction at the single gene level. Second, the genome is organized into functionally distinct topologically associated domains (TADs), from 100 kilo-bases to 5 mega-bases, encompassing multiple genes and regulatory elements [50, 52, 53]. TAD boundaries act as insulators by preventing communication between regulatory elements on either side, and TADs are mostly invariant between different cell types, while loops within TADs (sub-TADs) are more tissue-specific [50, 52, 53]. Finally, interactions between TADs organize the genome into mega-base compartments comprising open gene-rich chromatin or closed gene-poor chromatin [54]. In LUSC, the expression correlation between *PD*-*L1* and Chr9p24 genes indicate the existence of a specific TAD architecture making *PD*-*L1*, but not *PD*-*L2*, expression largely dependent on enhancers shared with other Chr9p24 localized genes within this sub-TAD. One consequence is the possibility of *PD*-*L1* expression in a non-IFN stimulated tumor environment or despite genetic defects in the IFN signaling cascade. Thus, gene expression profiling of Chr9p24 could have potential as an IFN-γ signaling independent predictive biomarker for *PD*-*L1* expression with relevance for predicting responders to immunotherapy with PD-1/PD-L1 axis blockade in lung squamous cell carcinoma.

The limitations of this study are as follows. This was a retrospective study focusing on available gene expression data from TCGA NSCLC tumors and CCLE NSCLC cell lines. Gene expression data originated from different experimental platforms and raw data initially processed with different normalization strategies. Consequences related to potential data overfitting and lack of optimal data harmonization could negatively impair the validity of the subsequent comparisons. Nevertheless, the identified gene-signatures could be an important basis from which individual genes can be selected for further validation at mRNA and protein level in subsequent expression based analyses. In this line, the inclusion of further clinical data will be valuable to substantiate the usability of expression analyses of the hereby identified genes and gene-signatures as potential new clinical relevant response and resistance biomarkers for immunotherapy with PD-1/PD-L1 axis blockade in NSCLC.

## Conclusions

The analyses of this study revealed genes and gene signatures associated with *PD*-*L1* and *PD*-*L2* mRNA expression in NSCLC. The presented findings could possess importance in relation to a better understanding of the PD-1 checkpoint blockade-responsive biology and development of gene expression profile-based biomarkers for predicting clinical responses to immunotherapy.

## Additional files

**Additional file 1: Table S1.** Expression correlated genes for *PD-L1*, *PD-L2*, and both.

**Additional file 2: Fig. S1.** *PD*-*L1* and *PD*-*L2* expression correlated genes converge differently with *IRF1* and *IRF9* expression correlated genes in CCLE dataset Lung_NSC. **A**. Venn diagrams illustrating the number of genes with mRNA expression correlation in CCLE dataset (Lung_NSC, n = 114) using the GenomicScape portal for *PD*-*L1*, *PD*-*L2,* and *IRF1* (left panel), *PD*-*L1*, *PD*-*L2* and *IRF9* (central panel), and *IRF1* and *IRF9* (right panel). The criteria for significant expression correlation were Pearson correlation coefficient *r *≥ 0.3 or ≤ − 0.3, Spearman correlation coefficient *r* ≥ 0.4 or ≤ − 0.4, and all *P* values < 0.05. **B**. Venn diagrams illustrating the number of significant MSigDB hallmark gene sets for the genes in the Lung_NSC dataset having mRNA expression correlation with *PD*-*L1*, *PD*-*L2* and *IRF1* (left panel), *PD*-*L1*, *PD*-*L2* and *IRF9* (central panel), and *IRF1* and *IRF9* (right panel). Only selected genes and MSigDB hallmark gene sets are illustrated.

**Additional file 3: Table S2.** GSEA of *PD*-*L1* and *PD*-*L2* expression correlated genes in CCLE dataset (Lung_NSC) and TCGA datasets (LUAD and LUSC).

**Additional file 4: Table S3.** Expression correlation between *PD*-*L1*, *PD*-*L2*, IFN signaling pathway genes, and PD-L1 and PD-L2 cognate-receptors in CCLE dataset (Lung_NSC) and TCGA datasets (LUAD and LUSC).

**Additional file 5: Table S4.** Expression correlated genes for *IRF1* and *IRF9*.

**Additional file 6: Table S5.** GSEA of *IRF1* and *IRF9* expression correlated genes in CCLE dataset (Lung_NSC) and TCGA datasets (LUAD and LUSC).

**Additional file 7: Table S6.** Differences in expression in TCGA datasets (LUAD and LUSC) versus normal paired tissue from GTEx and TCGA.

**Additional file 8: Fig. S2.** *PD*-*L1* and *PD*-*L2* expression correlated genes converge differently with *IRF1* and *IRF9* expression correlated genes in TCGA datasets (LUAD and LUSC). **A**. Venn diagrams illustrating the number of genes in LUAD (n = 517) having mRNA expression correlation with *PD*-*L1*, *PD*-*L2* and *IRF1* (left panel), *PD*-*L1*, *PD*-*L2* and *IRF9* (central panel), and *IRF1* and *IRF9* (right panel). The criteria for significant expression correlation are: Pearson correlation coefficient r ≥ 0.3 or ≤ − 0.3, Spearman correlation coefficient *r* ≥ 0.4 or ≤ − 0.4, and all *P* values < 0.05. The analysis was performed using cBioPortal. **B**. Venn diagrams illustrating the number of significant MSigDB hallmark gene sets for the genes in the LUAD dataset having mRNA expression correlation with *PD*-*L1*, *PD*-*L2* and *IRF1* (left panel), *PD*-*L1*, *PD*-*L2* and *IRF9* (central panel), and *IRF1* and *IRF9* (right panel). **C**, **D**. Panels **C**, **D** are similar to panels **A**, **B** except that the LUSC dataset (n = 501) is analyzed in panels **C**, **D**. Only selected genes and MSigDB hallmark gene sets are illustrated.

**Additional file 9: Table S7.** Gene lists used for various analyses.

**Additional file 10: Table S8.** Gene lists representing various subsets of *PD*-*L1* and *PD*-*L2* expression correlated genes in CCLE dataset (Lung_NSC) and TCGA datasets (LUAD and LUSC).

**Additional file 11: Fig. S3.** Heat map analyses of *PD*-*L1* and *PD*-*L2* expression correlation gene signatures in TCGA datasets (LUAD and LUSC). **A**, **B**. Heat map analyses of mRNA expression Z-values in TCGA dataset LUSC **A** and LUAD **B** of gene signatures representing expression correlated genes with *PD*-*L1* and *PD*-*L2* across Lung_NSC, LUAD, and LUSC. Heat maps are sorted relative to the *PD*-*L1* mRNA expression level (upper panels) or *PD*-*L2* mRNA expression level (lower panels). Spearman and Pearson correlation coefficients *r* and corresponding *P* values for mRNA expression of signature genes and PD-L1 protein expression in LUAD (n = 365) and LUSC (n = 328) are shown to the right. **C**. Heat map analysis of mRNA expression Z-values for gene signatures representing genes expression correlated with *PD*-*L1* (upper panel) and *PD*-*L2* (lower panel) across LUAD and Lung_NSC. The heat map is sorted relative to *PD*-*L1* mRNA expression level (upper panels) and *PD*-*L2* mRNA expression level (lower panel). Spearman and Pearson correlation coefficients *r* and corresponding *P* values for mRNA expression of signature genes and PD-L1 protein expression in LUAD (n = 365) are shown to the right. Asterisks in the lower panel indicate genes also included in the analysis in the upper panel. Correlations assigned significant are shown in red. The criteria for significant expression correlation were Pearson correlation coefficient *r* ≥ 0.3 or ≤ − 0.3, Spearman correlation coefficient *r* ≥ 0.4 or ≤ − 0.4, and *P* values < 0.05. Abbreviations: IRF1cor, *IRF1* expression correlated genes; Pe, Pearson; r, correlation coefficient; Sp, Spearman.

**Additional file 12: Table S9.** GSEA for genomic localization of *PD*-*L1* and *PD*-*L2* expression correlated genes.

**Additional file 13: Fig.** **S4.** *PD*-*L1* expression correlated genes located at Chr9p24 clusters in LUSC. **A**, **B**. Unsupervised hierarchical cluster heat map analysis of mRNA expression Z-values from TCGA dataset LUSC **A** and LUAD **B** with a merged gene signature (n = 94) composed of *PD*-*L1* expression correlated genes in LUSC with localization to Chr9p24, the gene lists for immune cells from Garcia_Diaz et al. [21], and the gene list IFN signaling core composed of *IRF1*, *IRF9*, *STAT1*, *JAK1*, and *JAK2*. *PD*-*L1* expression correlated genes with Chr9p24 localization are highlighted.

## Abbreviations

BMP: bone morphogenic protein
BMPR I: BMP receptor type 1
BMPR II: BMP receptor type 2
C2-type: constant 2-type
CCLE: cancer cell line encyclopedia
CD80: cluster of differentiation 80
GSEA: gene set enrichment analysis
GTEx: genotype-tissue expression project
IFN: interferon
Ig-like: immunoglobulin-like
IL: interleukin
LUAD: TCGA lung adenocarcinoma
LUSC: TCGA lung squamous cell carcinoma
NK: natural killer cell
NSCLC: non-small-cell lung cancer
PD-1: programmed cell death protein-1
PD-L1: programmed cell death ligand-1
PD-L2: programmed cell death ligand-2
Pe: pearson
r: expression correlation coefficient
RGMB: repulsive guidance molecule B
RMA: robust multi-array average
RPKM: per million mapped reads
RSEM: RNA-sequencing by expectation–maximization
SHP-1/2: Src homology 2 domain-containing protein tyrosine phosphatase 1 and 2
Sp: spearman
TAD: topologically associated domain
TCGA: the cancer genome atlas
TPM: transcripts per million
TPS: tumor proportion score
V-type: variable-type

## Availability of data and materials

The data supporting the conclusions of this article are included in the article.

## References

[1] Chen Z, Fillmore CM, Hammerman PS, Kim CF, Wong KK (2014). Non-small-cell lung cancers: a heterogeneous set of diseases. Nat Rev Cancer.

[2] Meng X, Liu Y, Zhang J, Teng F, Xing L, Yu J (2017). PD-1/PD-L1 checkpoint blockades in non-small cell lung cancer: new development and challenges. Cancer Lett.

[3] Remon J, Chaput N, Planchard D (2016). Predictive biomarkers for programmed death-1/programmed death ligand immune checkpoint inhibitors in non-small cell lung cancer. Curr Opin Oncol.

[4] Borghaei H, Paz-Ares L, Horn L, Spigel DR, Steins M, Ready NE (2015). Nivolumab versus docetaxel in advanced nonsquamous non-small-cell lung cancer. N Engl J Med.

[5] Brahmer J, Reckamp KL, Baas P, Crino L, Eberhardt WE, Poddubskaya E (2015). Nivolumab versus docetaxel in advanced squamous-cell non-small-cell lung cancer. N Engl J Med.

[6] Meyers DE, Bryan PM, Banerji S, Morris DG (2018). Targeting the PD-1/PD-L1 axis for the treatment of non-small-cell lung cancer. Curr Oncol..

[7] Sharpe AH, Wherry EJ, Ahmed R, Freeman GJ (2007). The function of programmed cell death 1 and its ligands in regulating autoimmunity and infection. Nat Immunol.

[8] Jin HT, Ahmed R, Okazaki T (2011). Role of PD-1 in regulating T-cell immunity. Curr Top Microbiol Immunol.

[9] Bally AP, Austin JW, Boss JM (2016). Genetic and epigenetic regulation of PD-1 expression. J Immunol..

[10] Francisco LM, Salinas VH, Brown KE, Vanguri VK, Freeman GJ, Kuchroo VK (2009). PD-L1 regulates the development, maintenance, and function of induced regulatory T cells. J Exp Med.

[11] Yamazaki T, Akiba H, Iwai H, Matsuda H, Aoki M, Tanno Y (2002). Expression of programmed death 1 ligands by murine T cells and APC. J Immunol..

[12] Dai S, Jia R, Zhang X, Fang Q, Huang L (2014). The PD-1/PD-Ls pathway and autoimmune diseases. Cell Immunol.

[13] Arasanz H, Gato-Canas M, Zuazo M, Ibanez-Vea M, Breckpot K, Kochan G (2017). PD1 signal transduction pathways in T cells. Oncotarget..

[14] Fife BT, Pauken KE (2011). The role of the PD-1 pathway in autoimmunity and peripheral tolerance. Ann N Y Acad Sci.

[15] He J, Hu Y, Hu M, Li B (2015). Development of PD-1/PD-L1 pathway in tumor immune microenvironment and treatment for non-small cell lung cancer. Sci Rep..

[16] Rozali EN, Hato SV, Robinson BW, Lake RA, Lesterhuis WJ (2012). Programmed death ligand 2 in cancer-induced immune suppression. Clin Dev Immunol.

[17] Butte MJ, Keir ME, Phamduy TB, Sharpe AH, Freeman GJ (2007). Programmed death-1 ligand 1 interacts specifically with the B7-1 costimulatory molecule to inhibit T cell responses. Immunity.

[18] Xiao Y, Yu S, Zhu B, Bedoret D, Bu X, Francisco LM (2014). RGMb is a novel binding partner for PD-L2 and its engagement with PD-L2 promotes respiratory tolerance. J Exp Med.

[19] Nie X, Chen W, Zhu Y, Huang B, Yu W, Wu Z (2017). B7-DC (PD-L2) costimulation of CD4(+) T-helper 1 response via RGMb. Cell Mol Immunol.

[20] Chen L, Han X (2015). Anti-PD-1/PD-L1 therapy of human cancer: past, present, and future. J Clin Invest..

[21] Garcia-Diaz A, Shin DS, Moreno BH, Saco J, Escuin-Ordinas H, Rodriguez GA (2017). Interferon receptor signaling pathways regulating PD-L1 and PD-L2 expression. Cell Rep..

[22] Pitt JM, Vetizou M, Daillere R, Roberti MP, Yamazaki T, Routy B (2016). Resistance mechanisms to immune-checkpoint blockade in cancer: tumor-intrinsic and -extrinsic factors. Immunity.

[23] Wang A, Wang HY, Liu Y, Zhao MC, Zhang HJ, Lu ZY (2015). The prognostic value of PD-L1 expression for non-small cell lung cancer patients: a meta-analysis. Eur J Surg Oncol.

[24] Zhang M, Li G, Wang Y, Wang Y, Zhao S, Haihong P (2017). PD-L1 expression in lung cancer and its correlation with driver mutations: a meta-analysis. Sci Rep..

[25] Abdel-Rahman O (2016). Correlation between PD-L1 expression and outcome of NSCLC patients treated with anti-PD-1/PD-L1 agents: a meta-analysis. Crit Rev Oncol Hematol.

[26] Passiglia F, Bronte G, Bazan V, Natoli C, Rizzo S, Galvano A (2016). PD-L1 expression as predictive biomarker in patients with NSCLC: a pooled analysis. Oncotarget..

[27] Garon EB, Rizvi NA, Hui R, Leighl N, Balmanoukian AS, Eder JP (2015). Pembrolizumab for the treatment of non-small-cell lung cancer. N Engl J Med.

[28] Hui R, Garon EB, Goldman JW, Leighl NB, Hellmann MD, Patnaik A (2017). Pembrolizumab as first-line therapy for patients with PD-L1-positive advanced non-small cell lung cancer: a phase 1 trial. Ann Oncol.

[29] Reck M, Rodriguez-Abreu D, Robinson AG, Hui R, Csoszi T, Fulop A (2016). Pembrolizumab versus chemotherapy for PD-L1-positive non-small-cell lung cancer. N Engl J Med.

[30] Heigener DF, Reck M (2018). Advanced non-small cell lung cancer: the role of PD-L1 inhibitors. J Thorac Dis..

[31] Pillai RN, Behera M, Owonikoko TK, Kamphorst AO, Pakkala S, Belani CP (2018). Comparison of the toxicity profile of PD-1 versus PD-L1 inhibitors in non-small cell lung cancer: a systematic analysis of the literature. Cancer.

[32] Zhang M, Feng D, Jing J, Liu H, Zhao S, Zhang Q (2017). PD-L1 protein expression in non-small cell lung cancer based on different immunohistochemical antibodies. J Thorac Dis..

[33] Barretina J, Caponigro G, Stransky N, Venkatesan K, Margolin AA, Kim S (2012). The cancer cell line encyclopedia enables predictive modelling of anticancer drug sensitivity. Nature.

[34] Weinstein JN, Collisson EA, Mills GB, Shaw KR, Ozenberger BA, Cancer Genome Atlas Research N (2013). The cancer genome atlas pan-cancer analysis project. Nat Genet..

[35] Consortium GT (2013). The genotype-tissue expression (GTEx) project. Nat Genet.

[36] Liberzon A, Birger C, Thorvaldsdottir H, Ghandi M, Mesirov JP, Tamayo P (2015). The molecular signatures database (MSigDB) hallmark gene set collection. Cell Syst..

[37] Subramanian A, Tamayo P, Mootha VK, Mukherjee S, Ebert BL, Gillette MA (2005). Gene set enrichment analysis: a knowledge-based approach for interpreting genome-wide expression profiles. Proc Natl Acad Sci USA.

[38] Chae YK, Chang S, Ko T, Anker J, Agte S, Iams W (2018). Epithelial-mesenchymal transition (EMT) signature is inversely associated with T-cell infiltration in non-small cell lung cancer (NSCLC). Sci Rep..

[39] Chen L, Gibbons DL, Goswami S, Cortez MA, Ahn YH, Byers LA (2014). Metastasis is regulated via microRNA-200/ZEB1 axis control of tumour cell PD-L1 expression and intratumoral immunosuppression. Nat Commun..

[40] Ayers M, Lunceford J, Nebozhyn M, Murphy E, Loboda A, Kaufman DR (2017). IFN-gamma-related mRNA profile predicts clinical response to PD-1 blockade. J Clin Invest..

[41] Shin DS, Zaretsky JM, Escuin-Ordinas H, Garcia-Diaz A, Hu-Lieskovan S, Kalbasi A (2017). Primary resistance to PD-1 blockade mediated by JAK1/2 mutations. Cancer Discov.

[42] Zaretsky JM, Garcia-Diaz A, Shin DS, Escuin-Ordinas H, Hugo W, Hu-Lieskovan S (2016). Mutations associated with acquired resistance to PD-1 blockade in melanoma. N Engl J Med.

[43] Horn S, Leonardelli S, Sucker A, Schadendorf D, Griewank KG, Paschen A (2018). Tumor CDKN2A-associated JAK2 loss and susceptibility to immunotherapy resistance. J Natl Cancer Inst.

[44] Ikeda S, Okamoto T, Okano S, Umemoto Y, Tagawa T, Morodomi Y (2016). PD-L1 is upregulated by simultaneous amplification of the PD-L1 and JAK2 genes in non-small cell lung cancer. J Thorac Oncol..

[45] Harris T, Pan Q, Sironi J, Lutz D, Tian J, Sapkar J (2011). Both gene amplification and allelic loss occur at 14q13.3 in lung cancer. Clin Cancer Res..

[46] Luk C, Tsao MS, Bayani J, Shepherd F, Squire JA (2001). Molecular cytogenetic analysis of non-small cell lung carcinoma by spectral karyotyping and comparative genomic hybridization. Cancer Genet Cytogenet.

[47] Pei J, Balsara BR, Li W, Litwin S, Gabrielson E, Feder M (2001). Genomic imbalances in human lung adenocarcinomas and squamous cell carcinomas. Genes Chromosomes Cancer.

[48] Petersen I, Bujard M, Petersen S, Wolf G, Goeze A, Schwendel A (1997). Patterns of chromosomal imbalances in adenocarcinoma and squamous cell carcinoma of the lung. Cancer Res.

[49] Weir BA, Woo MS, Getz G, Perner S, Ding L, Beroukhim R (2007). Characterizing the cancer genome in lung adenocarcinoma. Nature.

[50] Achinger-Kawecka J, Clark SJ (2017). Disruption of the 3D cancer genome blueprint. Epigenomics..

[51] Dekker J, Mirny L (2016). The 3D genome as moderator of chromosomal communication. Cell.

[52] Dixon JR, Selvaraj S, Yue F, Kim A, Li Y, Shen Y (2012). Topological domains in mammalian genomes identified by analysis of chromatin interactions. Nature.

[53] Krijger PH, de Laat W (2016). Regulation of disease-associated gene expression in the 3D genome. Nat Rev Mol Cell Biol.

[54] Lieberman-Aiden E, van Berkum NL, Williams L, Imakaev M, Ragoczy T, Telling A (2009). Comprehensive mapping of long-range interactions reveals folding principles of the human genome. Science.

