# Supplementary figures and images for: PD-L1 and PD-L2 expression correlated genes in non-small-cell lung cancer
Source: Cancer Commun (Lond). 2019 Jun 3;39:30. doi: 10.1186/s40880-019-0376-6 (PMC6545701; doi:10.1186/s40880-019-0376-6)

A

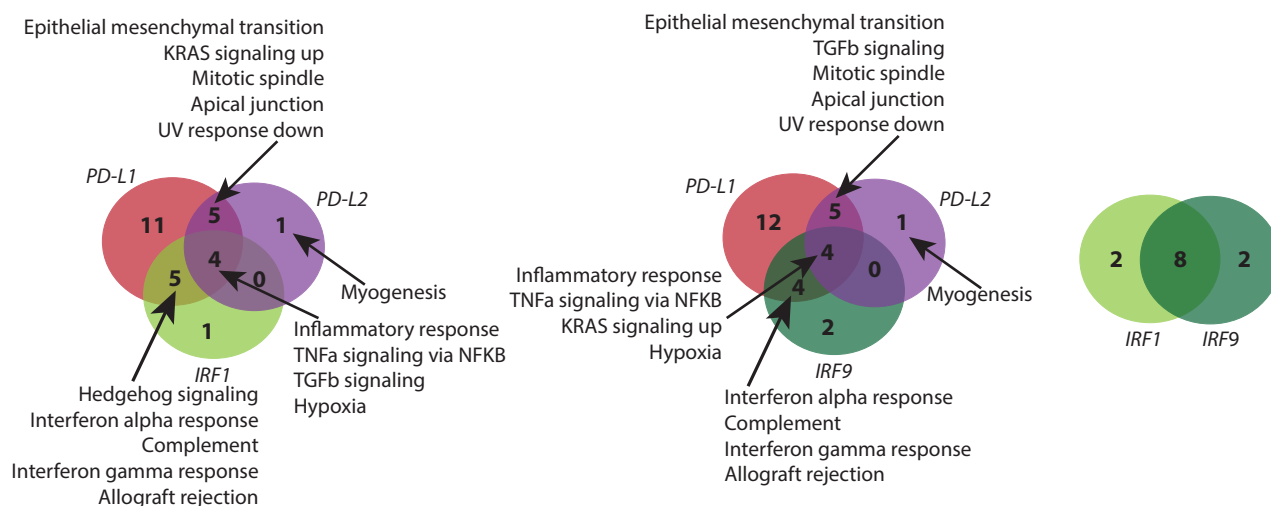

B

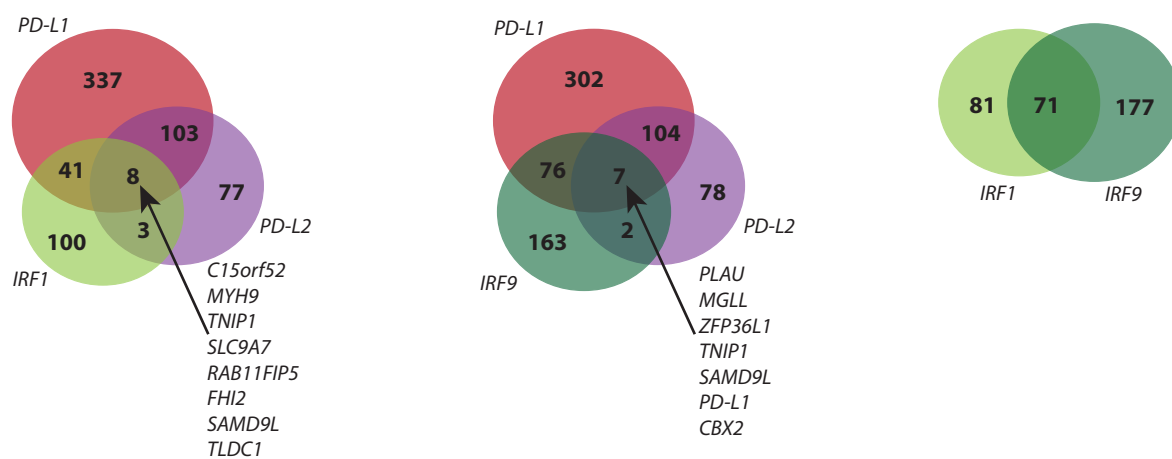

Supplementary Figure 1

Supplement: Supplementary file 2 — Additional file 2: Fig. S1. PD-L1 and PD-L2 expression correlated genes converge differently with IRF1 and IRF9 expression correlated genes in CCLE dataset Lung_NSC. A. Venn diagrams illustrating the number of genes with mRNA expression correlation in CCLE dataset (Lung_NSC, n = 114) using the GenomicScape portal for PD-L1, PD-L2, and IRF1 (left panel), PD-L1, PD-L2 and IRF9 (central panel), and IRF1 and IRF9 (right panel). The criteria for significant expression correlation were Pearson correlation coefficient r ≥ 0.3 or ≤ − 0.3, Spearman correlation coefficient r ≥ 0.4 or ≤ − 0.4, and all P values < 0.05. B. Venn diagrams illustrating the number of significant MSigDB hallmark gene sets for the genes in the Lung_NSC dataset having mRNA expression correlation with PD-L1, PD-L2 and IRF1 (left panel), PD-L1, PD-L2 and IRF9 (central panel), and IRF1 and IRF9 (right panel). Only selected genes and MSigDB hallmark gene sets are illustrated. [file 40880_2019_376_MOESM2_ESM.pdf]

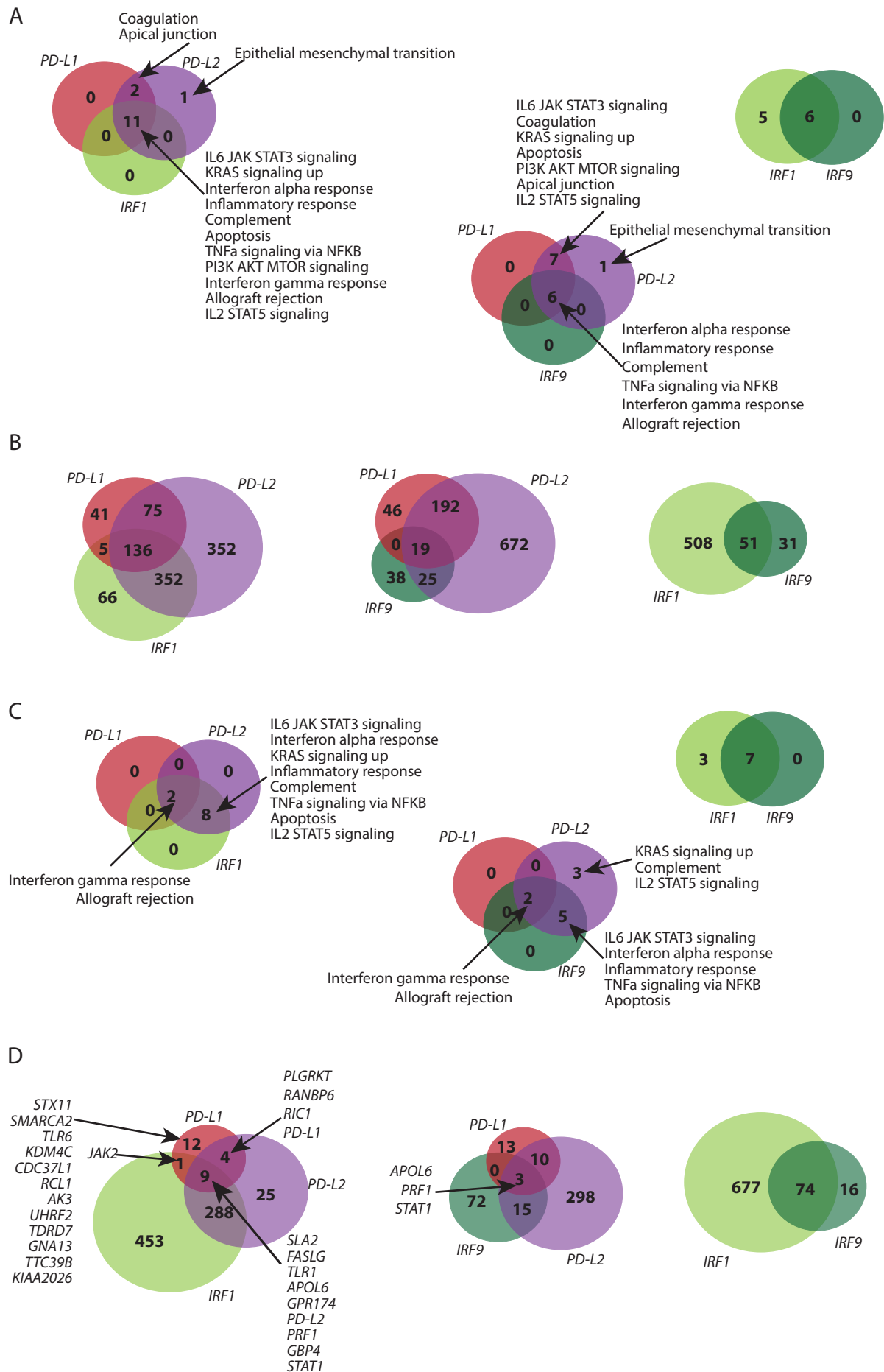

Supplementary Figure 2

Supplement: Supplementary file 8 — Additional file 8: Fig. S2. PD-L1 and PD-L2 expression correlated genes converge differently with IRF1 and IRF9 expression correlated genes in TCGA datasets (LUAD and LUSC). A. Venn diagrams illustrating the number of genes in LUAD (n = 517) having mRNA expression correlation with PD-L1, PD-L2 and IRF1 (left panel), PD-L1, PD-L2 and IRF9 (central panel), and IRF1 and IRF9 (right panel). The criteria for significant expression correlation are: Pearson correlation coefficient r ≥ 0.3 or ≤ − 0.3, Spearman correlation coefficient r ≥ 0.4 or ≤ − 0.4, and all P values < 0.05. The analysis was performed using cBioPortal. B. Venn diagrams illustrating the number of significant MSigDB hallmark gene sets for the genes in the LUAD dataset having mRNA expression correlation with PD-L1, PD-L2 and IRF1 (left panel), PD-L1, PD-L2 and IRF9 (central panel), and IRF1 and IRF9 (right panel). C, D. Panels C, D are similar to panels A, B except that the LUSC dataset (n = 501) is analyzed in panels C, D. Only selected genes and MSigDB hallmark gene sets are illustrated. [file 40880_2019_376_MOESM8_ESM.pdf]

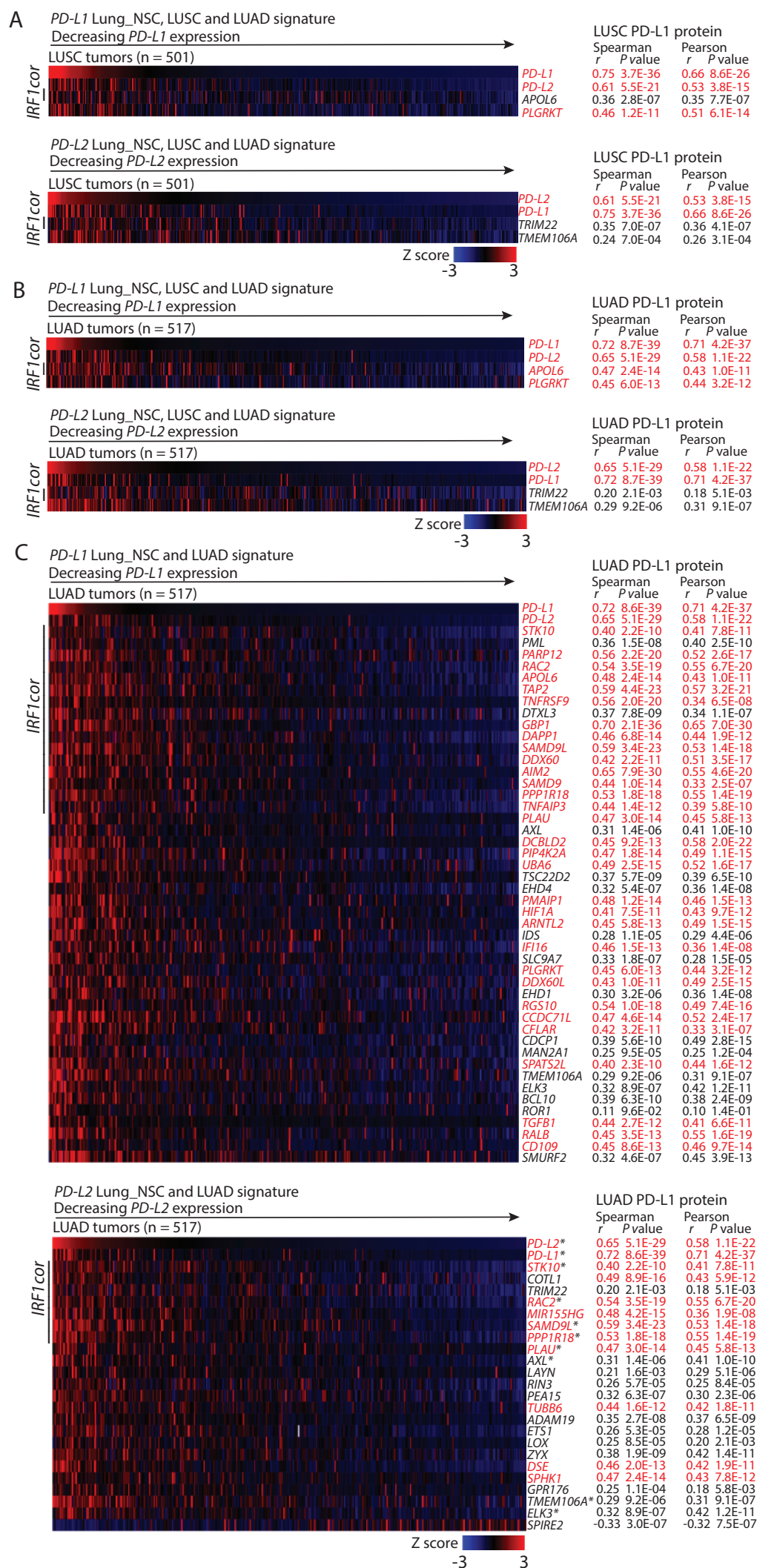

Supplementary Figure 3

Supplement: Supplementary file 11 — Additional file 11: Fig. S3. Heat map analyses of PD-L1 and PD-L2 expression correlation gene signatures in TCGA datasets (LUAD and LUSC). A, B. Heat map analyses of mRNA expression Z-values in TCGA dataset LUSC A and LUAD B of gene signatures representing expression correlated genes with PD-L1 and PD-L2 across Lung_NSC, LUAD, and LUSC. Heat maps are sorted relative to the PD-L1 mRNA expression level (upper panels) or PD-L2 mRNA expression level (lower panels). Spearman and Pearson correlation coefficients r and corresponding P values for mRNA expression of signature genes and PD-L1 protein expression in LUAD (n = 365) and LUSC (n = 328) are shown to the right. C. Heat map analysis of mRNA expression Z-values for gene signatures representing genes expression correlated with PD-L1 (upper panel) and PD-L2 (lower panel) across LUAD and Lung_NSC. The heat map is sorted relative to PD-L1 mRNA expression level (upper panels) and PD-L2 mRNA expression level (lower panel). Spearman and Pearson correlation coefficients r and corresponding P values for mRNA expression of signature genes and PD-L1 protein expression in LUAD (n = 365) are shown to the right. Asterisks in the lower panel indicate genes also included in the analysis in the upper panel. Correlations assigned significant are shown in red. The criteria for significant expression correlation were Pearson correlation coefficient r ≥ 0.3 or ≤ − 0.3, Spearman correlation coefficient r ≥ 0.4 or ≤ − 0.4, and P values < 0.05. Abbreviations: IRF1cor, IRF1 expression correlated genes; Pe, Pearson; r, correlation coefficient; Sp, Spearman. [file 40880_2019_376_MOESM11_ESM.pdf]

A

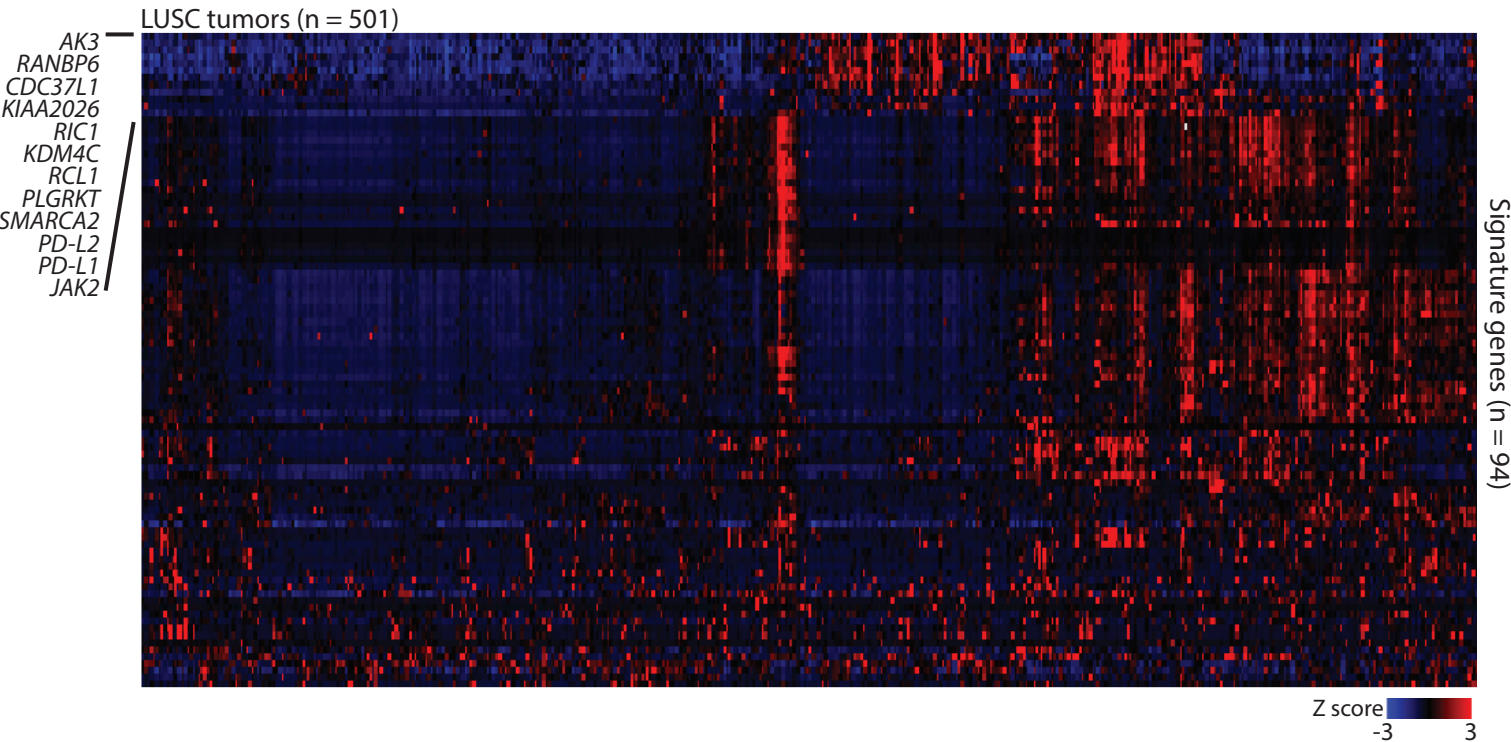

B

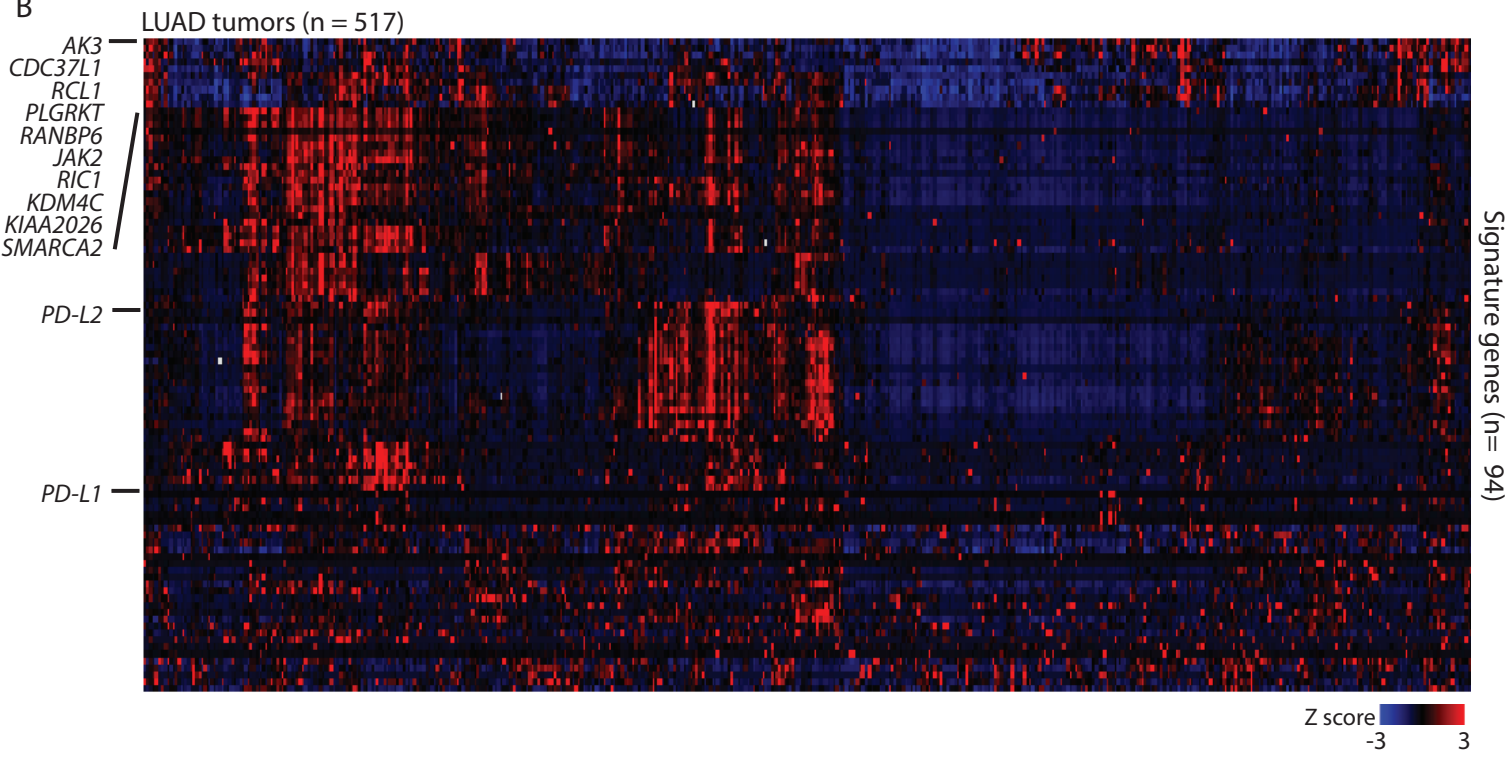

Supplementary Figure 4

Supplement: Supplementary file 13 — Additional file 13: Fig. S4. PD-L1 expression correlated genes located at Chr9p24 clusters in LUSC. A, B. Unsupervised hierarchical cluster heat map analysis of mRNA expression Z-values from TCGA dataset LUSC A and LUAD B with a merged gene signature (n = 94) composed of PD-L1 expression correlated genes in LUSC with localization to Chr9p24, the gene lists for immune cells from Garcia_Diaz et al. [21], and the gene list IFN signaling core composed of IRF1, IRF9, STAT1, JAK1, and JAK2. PD-L1 expression correlated genes with Chr9p24 localization are highlighted. [file 40880_2019_376_MOESM13_ESM.pdf]
