# Supplementary material for: PD-L1 and PD-L2 expression correlated genes in non-small-cell lung cancer
Source: Cancer Commun (Lond). 2019 Jun 3;39:30. doi: 10.1186/s40880-019-0376-6 (PMC6545701; doi:10.1186/s40880-019-0376-6)
Supplement: Supplementary file 3 — Additional file 3: Table S2. GSEA of PD-L1 and PD-L2 expression correlated genes in CCLE dataset (Lung_NSC) and TCGA datasets (LUAD and LUSC). [file 40880_2019_376_MOESM3_ESM.docx]

**Table S2** GSEA of *PD-L1* and *PD-L2* expression correlated genes in CCLE dataset (Lung_NSC) and TCGA dataset (LUAD and LUSC).

| **MSigDB hallmark gene set** | **K** | **Description of gene set** | **k** | **k/K** | ***P*** | ***q*** |
| --- | --- | --- | --- | --- | --- | --- |
| **CCLE dataset (Lung_NSC)** | | | | | | |
| ***PD-L1* expression correlated genes (n = 489)** | | | | | | |
| TNFa signaling via NFKB | 200 | Genes regulated by NF-kB in response to TNF [GeneID=7124]. | 33 | 0.17 | 7.49E-30 | 3.75E-28 |
| KRAS signaling up | 200 | Genes up-regulated by KRAS activation. | 25 | 0.13 | 7.31E-20 | 1.83E-18 |
| Hypoxia | 200 | Genes up-regulated in response to low oxygen levels (hypoxia). | 24 | 0.12 | 1.06E-18 | 1.77E-17 |
| Epithelial mesenchymal transition | 200 | Genes defining epithelial-mesenchymal transition, as in wound healing, fibrosis and metastasis. | 22 | 0.11 | 1.94E-16 | 1.62E-15 |
| Inflammatory response | 200 | Genes defining inflammatory response. | 22 | 0.11 | 1.94E-16 | 1.62E-15 |
| Interferon gamma response | 200 | Genes up-regulated in response to IFNG [GeneID=3458]. | 22 | 0.11 | 1.94E-16 | 1.62E-15 |
| Interferon alpha response | 97 | Genes up-regulated in response to alpha interferon proteins. | 14 | 0.14 | 1.38E-12 | 9.84E-12 |
| Apoptosis | 161 | Genes mediating programmed cell death (apoptosis) by activation of caspases. | 16 | 0.10 | 1.21E-11 | 7.58E-11 |
| Apical junction | 200 | Genes encoding components of apical junction complex. | 17 | 0.09 | 3.40E-11 | 1.89E-10 |
| Estrogen response early | 200 | Genes defining early response to estrogen. | 16 | 0.08 | 3.17E-10 | 1.44E-09 |
| Glycolysis | 200 | Genes encoding proteins involved in glycolysis and gluconeogenesis. | 16 | 0.08 | 3.17E-10 | 1.44E-09 |
| UV response down | 144 | Genes down-regulated in response to ultraviolet (UV) radiation. | 12 | 0.08 | 3.33E-08 | 1.39E-07 |
| Estrogen response late | 200 | Genes defining late response to estrogen. | 13 | 0.07 | 1.69E-07 | 6.51E-07 |
| Allograft rejection | 200 | Genes up-regulated during transplant rejection. | 12 | 0.06 | 1.18E-06 | 3.68E-06 |
| Complement | 200 | Genes encoding components of the complement system, which is part of the innate immune system. | 12 | 0.06 | 1.18E-06 | 3.68E-06 |
| Heme metabolism | 200 | Genes involved in metabolism of heme (a cofactor consisting of iron and porphyrin) and erythroblast differentiation. | 12 | 0.06 | 1.18E-06 | 3.68E-06 |
| TGFb signaling | 54 | Genes up-regulated in response to TGFB1 [GeneID=7040]. | 7 | 0.13 | 1.27E-06 | 3.74E-06 |
| Hedgehog signaling | 36 | Genes up-regulated by activation of hedgehog signaling. | 6 | 0.17 | 1.62E-06 | 4.51E-06 |
| Mitotic spindle | 200 | Genes important for mitotic spindle assembly. | 11 | 0.06 | 7.52E-06 | 1.98E-05 |
| Notch signaling | 32 | Genes up-regulated by activation of Notch signaling. | 5 | 0.16 | 1.72E-05 | 4.30E-05 |
| IL2 STAT5 signaling | 200 | Genes up-regulated by STAT5 in response to IL2 stimulation. | 10 | 0.05 | 4.39E-05 | 1.04E-04 |
| G2M checkpoint | 200 | Genes involved in the G2/M checkpoint, as in progression through the cell division cycle. | 9 | 0.05 | 2.32E-04 | 4.63E-04 |
| Oxidative phosphorylation | 200 | Genes encoding proteins involved in oxidative phosphorylation. | 9 | 0.05 | 2.32E-04 | 4.63E-04 |
| P53 pathway | 200 | Genes involved in p53 pathways and networks. | 9 | 0.05 | 2.32E-04 | 4.63E-04 |
| ***PD-L2* expression correlated genes (n = 191)** | | | | | | |
| Epithelial mesenchymal transition | 200 | Genes defining epithelial-mesenchymal transition, as in wound healing, fibrosis and metastasis. | 16 | 0.08 | 1.41E-16 | 7.05E-15 |
| Hypoxia | 200 | Genes up-regulated in response to low oxygen levels (hypoxia). | 14 | 0.07 | 7.51E-14 | 1.88E-12 |
| TNFa signaling via NFKB | 200 | Genes regulated by NF-kB in response to TNF [GeneID=7124]. | 13 | 0.07 | 1.54E-12 | 2.57E-11 |
| Apical junction | 200 | Genes encoding components of apical junction complex. | 10 | 0.05 | 7.81E-09 | 7.81E-08 |
| KRAS signaling up | 200 | Genes up-regulated by KRAS activation. | 10 | 0.05 | 7.81E-09 | 7.81E-08 |
| UV response down | 144 | Genes down-regulated in response to ultraviolet (UV) radiation. | 7 | 0.05 | 1.76E-06 | 1.47E-05 |
| TGFb signaling | 54 | Genes up-regulated in response to TGFB1 [GeneID=7040]. | 5 | 0.09 | 2.36E-06 | 1.69E-05 |
| Inflammatory response | 200 | Genes defining inflammatory response. | 7 | 0.04 | 1.52E-05 | 7.61E-05 |
| Mitotic spindle | 200 | Genes important for mitotic spindle assembly. | 7 | 0.04 | 1.52E-05 | 7.61E-05 |
| Myogenesis | 200 | Genes involved in development of skeletal muscle (myogenesis). | 7 | 0.04 | 1.52E-05 | 7.61E-05 |
| **TCGA dataset (LUAD)** | | | | | | |
| ***PD-L1* expression correlated genes (n = 257)** | | | | | | |
| Interferon gamma response | 200 | Genes up-regulated in response to IFNG [GeneID=3458]. | 42 | 0.21 | 1.57E-55 | 7.83E-54 |
| Allograft rejection | 200 | Genes up-regulated during transplant rejection. | 31 | 0.16 | 1.12E-36 | 2.81E-35 |
| Interferon alpha response | 97 | Genes up-regulated in response to alpha interferon proteins. | 22 | 0.23 | 3.20E-30 | 5.33E-29 |
| Inflammatory response | 200 | Genes defining inflammatory response. | 26 | 0.13 | 8.09E-29 | 1.01E-27 |
| Complement | 200 | Genes encoding components of the complement system, which is part of the innate immune system. | 22 | 0.11 | 6.60E-23 | 6.60E-22 |
| IL6, JAK, STAT3 Signaling | 87 | Genes up-regulated by IL6 [GeneID=3569] via STAT3 [GeneID=6774], e.g., during acute phase response. | 14 | 0.16 | 2.07E-17 | 1.72E-16 |
| TNFa signaling via NFKB | 200 | Genes regulated by NF-kB in response to TNF [GeneID=7124]. | 16 | 0.08 | 8.99E-15 | 6.42E-14 |
| IL2 STAT5 signaling | 200 | Genes up-regulated by STAT5 in response to IL2 stimulation. | 12 | 0.06 | 6.02E-10 | 3.35E-09 |
| KRAS signaling up | 200 | Genes up-regulated by KRAS activation. | 12 | 0.06 | 6.02E-10 | 3.35E-09 |
| Apoptosis | 161 | Genes mediating programmed cell death (apoptosis) by activation of caspases. | 10 | 0.06 | 1.20E-08 | 5.99E-08 |
| Apical junction | 200 | Genes encoding components of apical junction complex. | 8 | 0.04 | 9.55E-06 | 4.34E-05 |
| Coagulation | 138 | Genes encoding components of blood coagulation system; also up-regulated in platelets. | 6 | 0.04 | 8.02E-05 | 3.34E-04 |
| PI3K, AKT, MTOR signaling | 105 | Genes up-regulated by activation of the PI3K/AKT/mTOR pathway. | 5 | 0.05 | 2.09E-04 | 8.05E-04 |
| ***PD-L2* expression correlated genes (n = 914)** | | | | | | |
| Allograft rejection | 200 | Genes up-regulated during transplant rejection. | 96 | 0.48 | 4.07E-111 | 2.04E-109 |
| Interferon gamma response | 200 | Genes up-regulated in response to IFNG [GeneID=3458]. | 87 | 0.44 | 7.65E-96 | 1.91E-94 |
| Inflammatory response | 200 | Genes defining inflammatory response. | 61 | 0.31 | 4.49E-56 | 7.48E-55 |
| Interferon alpha response | 97 | Genes up-regulated in response to alpha interferon proteins. | 40 | 0.41 | 2.52E-43 | 3.15E-42 |
| Complement | 200 | Genes encoding components of the complement system, which is part of the innate immune system. | 46 | 0.23 | 2.27E-36 | 2.27E-35 |
| IL6, JAK, STAT3 Signaling | 87 | Genes up-regulated by IL6 [GeneID=3569] via STAT3 [GeneID=6774], e.g., during acute phase response. | 30 | 0.34 | 5.16E-30 | 4.30E-29 |
| KRAS signaling up | 200 | Genes up-regulated by KRAS activation. | 39 | 0.20 | 4.03E-28 | 2.88E-27 |
| TNFa signaling via NFKB | 200 | Genes regulated by NF-kB in response to TNF [GeneID=7124]. | 38 | 0.19 | 5.36E-27 | 3.35E-26 |
| IL2 STAT5 signaling | 200 | Genes up-regulated by STAT5 in response to IL2 stimulation. | 36 | 0.18 | 8.56E-25 | 4.76E-24 |
| Apoptosis | 161 | Genes mediating programmed cell death (apoptosis) by activation of caspases. | 20 | 0.12 | 2.73E-11 | 1.36E-10 |
| Epithelial mesenchymal transition | 200 | Genes defining epithelial-mesenchymal transition, as in wound healing, fibrosis and metastasis. | 20 | 0.10 | 1.36E-09 | 6.19E-09 |
| Apical junction | 200 | Genes encoding components of apical junction complex. | 19 | 0.01 | 8.18E-09 | 3.41E-08 |
| PI3K, AKT, MTOR signaling | 105 | Genes up-regulated by activation of the PI3K/AKT/mTOR pathway. | 13 | 0.12 | 8.35E-08 | 3.21E-07 |
| Coagulation | 138 | Genes encoding components of blood coagulation system; also up-regulated in platelets. | 11 | 0.08 | 5.80E-05 | 2.07E-04 |
| **TCGA dataset (LUSC)** | | | | | | |
| ***PD-L1* expression correlated genes (n = 26)** | | | | | | |
| Allograft rejection | 200 | Genes up-regulated during transplant rejection. | 6 | 0.03 | 7.95E-10 | 1.99E-08 |
| Interferon gamma response | 200 | Genes up-regulated in response to IFNG [GeneID=3458]. | 6 | 0.03 | 7.95E-10 | 1.99E-08 |
| ***PD-L2* expression correlated genes (n = 326)** | | | | | | |
| Allograft rejection | 200 | Genes up-regulated during transplant rejection. | 55 | 0.28 | 2.71E-72 | 1.35E-70 |
| Interferon gamma response | 200 | Genes up-regulated in response to IFNG [GeneID=3458]. | 40 | 0.20 | 1.92E-46 | 4.81E-45 |
| Inflammatory response | 200 | Genes defining inflammatory response. | 29 | 0.15 | 1.31E-29 | 2.18E-28 |
| KRAS signaling up | 200 | Genes up-regulated by KRAS activation. | 23 | 0.12 | 2.36E-21 | 2.95E-20 |
| Complement | 200 | Genes encoding components of the complement system, which is part of the innate immune system. | 20 | 0.10 | 1.62E-17 | 1.62E-16 |
| IL6, JAK, STAT3 Signaling | 87 | Genes up-regulated by IL6 [GeneID=3569] via STAT3 [GeneID=6774], e.g., during acute phase response. | 13 | 0.15 | 3.89E-14 | 3.24E-13 |
| Interferon alpha response | 97 | Genes up-regulated in response to alpha interferon proteins. | 13 | 0.13 | 1.67E-13 | 1.19E-12 |
| IL2 STAT5 signaling | 200 | Genes up-regulated by STAT5 in response to IL2 stimulation. | 16 | 0.08 | 9.26E-13 | 5.79E-12 |
| TNFA signaling via NFKB | 200 | Genes regulated by NF-kB in response to TNF [GeneID=7124]. | 11 | 0.06 | 1.67E-07 | 9.30E-07 |
| Apoptosis | 161 | Genes mediating programmed cell death (apoptosis) by activation of caspases. | 7 | 0.04 | 1.34E-04 | 6.72E-04 |

K, number of genes in gene-set; k, number of genes in overlap. *P*, *P* value; *q*, q value for FDR. Cut off FDR q value < 1E-03.
