# Supplementary material for: PD-L1 and PD-L2 expression correlated genes in non-small-cell lung cancer
Source: Cancer Commun (Lond). 2019 Jun 3;39:30. doi: 10.1186/s40880-019-0376-6 (PMC6545701; doi:10.1186/s40880-019-0376-6)
Supplement: Supplementary file 4 — Additional file 4: Table S3. Expression correlation between PD-L1, PD-L2, IFN signaling pathway genes, and PD-L1 and PD-L2 cognate-receptors in CCLE dataset (Lung_NSC) and TCGA datasets (LUAD and LUSC). [file 40880_2019_376_MOESM4_ESM.docx]

**Table S3** Expression correlation between *PD-L1*, *PD-L2*, IFN signaling pathway genes, and PD-L1 and PD-L2 cognate-receptors in CCLE dataset (Lung_NSC) and TCGA dataset (LUAD and LUSC).

|  |  | ***PD-L1* mRNA** | | | ***PD-L2* mRNA** | | | ***IRF1* mRNA** | | | ***IRF9* mRNA** | | | **PD-L1 protein** | |
| --- | --- | --- | --- | --- | --- | --- | --- | --- | --- | --- | --- | --- | --- | --- | --- |
|  |  | **CCLE dataset** | **TCGA**  **dataset** | | **CCLE dataset** | **TCGA**  **dataset** | | **CCLE dataset** | **TCGA**  **dataset** | | **CCLE dataset** | **TCGA**  **dataset** | | **TCGA**  **dataset** | |
| **mRNA** | **Correlation** | **Lung-NSC** | **LUAD** | **LUSC** | **Lung-NSC** | **LUAD** | **LUSC** | **Lung-NSC** | **LUAD** | **LUSC** | **Lung-NSC** | **LUAD** | **LUSC** | **LUAD** | **LUSC** |
| ***IFNG*** | Pearson *r* | -0.10 | 0.32 | 0.31 | -0.13 | 0.49 | 0.30 | -0.05 | 0.63 | 0.46 | -0.10 | 0.17 | 0.25 | 0.44 | 0.44 |
|  | Spearman *r* | -0.11 | 0.48 | 0.35 | -0.02 | 0.53 | 0.50 | -0.06 | 0.70 | 0.70 | -0.14 | 0.32 | 0.38 | 0.58 | 0.50 |
| ***IFNGR1*** | Pearson *r* | -0.09 | 0.13 | 0.03 | -0.12 | 0.44 | 0.23 | 0.29 | 0.17 | 0.29 | 0.27 | -0.03 | 0.02 | 0.19 | 0.01 |
|  | Spearman *r* | -0.09 | 0.37 | 0.08 | 0.03 | 0.51 | 0.27 | 0.27 | 0.28 | 0.34 | 0.20 | 0.06 | 0.08 | 0.19 | 0.07 |
| ***IFNGR2*** | Pearson *r* | -0.05 | -0.06 | 0.05 | 0.03 | -0.03 | 0.10 | 0.18 | -0.08 | 0.20 | 0.31 | 0.12 | 0.11 | 0.10 | 0.08 |
|  | Spearman *r* | -0.02 | -0.06 | 0.06 | -0.17 | -0.06 | 0.17 | 0.22 | -0.06 | 0.22 | 0.25 | 0.14 | 0.03 | 0.13 | 0.16 |
| ***STAT1*** | Pearson *r* | 0.23 | 0.40 | 0.32 | 0.15 | 0.49 | 0.32 | 0.43 | 0.62 | 0.51 | 0.54 | 0.39 | 0.53 | 0.53 | 0.49 |
|  | Spearman *r* | 0.23 | 0.49 | 0.36 | 0.17 | 0.53 | 0.48 | 0.42 | 0.59 | 0.63 | 0.46 | 0.50 | 0.59 | 0.60 | 0.43 |
| ***JAK1*** | Pearson *r* | 0.21 | 0.19 | 0.06 | 0.28 | 0.28 | 0.15 | 0.42 | 0.23 | 0.29 | 0.32 | -0.04 | -0.03 | 0.08 | 0.04 |
|  | Spearman *r* | 0.26 | 0.39 | 0.15 | 0.27 | 0.40 | 0.31 | 0.44 | 0.24 | 0.40 | 0.32 | 0.01 | 0.07 | 0.07 | 0.06 |
| ***JAK2*** | Pearson *r* | 0.39 | 0.38 | 0.43 | 0.38 | 0.66 | 0.31* | 0.43 | 0.55 | 0.34 | 0.37 | 0.09 | 0.07 | 0.37 | 0.46 |
|  | Spearman *r* | 0.38 | 0.66 | 0.59 | 0.35 | 0.75 | 0.73 | 0.30 | 0.58 | 0.62 | 0.34 | 0.18 | 0.27 | 0.42 | 0.51 |
| ***IRF1*** | Pearson *r* | 0.26 | 0.41 | 0.38 | 0.22 | 0.56 | 0.32 | 1 | 1 | 1 | 0.58 | 0.25 | 0.33 | 0.53 | 0.44 |
|  | Spearman *r* | 0.32 | 0.64 | 0.41* | 0.23 | 0.68 | 0.59 | 1 | 1 | 1 | 0.52 | 0.38 | 0.47 | 0.59 | 0.40 |
| ***IRF9*** | Pearson *r* | 0.42 | 0.09 | 0.23 | 0.11 | 0.10 | 0.15 | 0.58 | 0.25 | 0.33 | 1 | 1 | 1 | 0.25 | 0.16 |
|  | Spearman *r* | 0.44 | 0.23 | 0.20 | 0.15 | 0.22 | 0.29 | 0.52 | 0.38 | 0.47 | 1 | 1 | 1 | 0.34 | 0.18 |
| ***TYK2*** | Pearson *r* | 0.02 | 0.00 | 0.14 | 0.23 | -0.11 | 0.08 | 0.12 | 0.04 | 0.21 | 0.04 | 0.24 | 0.04 | 0.02 | 0.14 |
|  | Spearman *r* | -0.03 | 0.09 | 0.16 | 0.18 | -0.04 | 0.13 | 0.07 | 0.07 | 0.21 | 0.01 | 0.23 | 0.12 | 0.07 | 0.15 |
| ***STAT2*** | Pearson *r* | 0.19 | 0.01 | 0.13 | 0.21 | 0.10 | 0.14 | 0.27 | 0.29 | 0.19 | 0.51 | 0.38 | 0.41 | 0.22 | 0.19 |
|  | Spearman *r* | 0.25 | 0.16 | 0.17 | 0.11 | 0.13 | 0.27 | 0.24 | 0.25 | 0.32 | 0.56 | 0.38 | 0.48 | 0.21 | 0.23 |
| ***STAT3*** | Pearson *r* | -0.05 | -0.07 | 0.05 | -0.04 | -0.06 | 0.10 | 0.32 | -0.05 | 0.27 | 0.28 | -0.10 | -0.07 | -0.26 | 0.05 |
|  | Spearman *r* | 0.03 | -0.06 | 0.12 | 0.03 | -0.02 | 0.19 | 0.32 | -0.09 | 0.28 | 0.29 | -0.15 | 0.03 | -0.35 | 0.03 |
| ***IFNAR1*** | Pearson *r* | -0.03 | 0.00 | 0.03 | 0.02 | 0.09 | 0.17 | 0.01 | 0.01 | 0.24 | 0.25 | 0.04 | -0.02 | -0.01 | -0.05 |
|  | Spearman *r* | -0.03 | 0.20 | 0.15 | -0.08 | 0.22 | 0.34 | 0.03 | 0.05 | 0.27 | 0.27 | 0.07 | 0.08 | 0.01 | -0.06 |
| ***IFNAR2*** | Pearson *r* | -0.14 | 0.09 | 0.08 | -0.16 | 0.13 | 0.27 | 0.17 | 0.10 | 0.41 | 0.23 | 0.15 | 0.07 | 0.13 | 0.08 |
|  | Spearman *r* | -0.13 | 0.29 | 0.21 | -0.03 | 0.24 | 0.48 | 0.17 | 0.17 | 0.51 | 0.20 | 0.21 | 0.19 | 0.17 | 0.11 |
| ***PD-1*** | Pearson *r* | -0.05 | 0.41 | 0.20 | -0.03 | 0.50 | 0.28 | 0.02 | 0.44 | 0.49 | -0.06 | 0.20 | 0.19 | 0.50 | 0.33 |
|  | Spearman *r* | -0.05 | 0.56 | 0.34 | -0.05 | 0.62 | 0.54 | -0.03 | 0.68 | 0.59 | -0.09 | 0.35 | 0.35 | 0.57 | 0.37 |
| ***CD80*** | Pearson *r* | 0.00 | 0.46 | 0.28 | -0.01 | 0.66 | 0.47 | -0.09 | 0.46 | 0.40 | 0.02 | 0.18 | 0.13 | 0.43 | 0.29 |
|  | Spearman *r* | 0.02 | 0.64 | 0.40 | 0.06 | 0.76 | 0.71 | -0.13 | 0.67 | 0.50 | 0.01 | 0.31 | 0.25 | 0.49 | 0.39 |
| ***RGMB*** | Pearson *r* | 0.33 | 0.00 | 0.04 | 0.58 | 0.03 | 0.02 | 0.14 | -0.09 | -0.15 | 0.07 | -0.13 | 0.00 | -0.15 | 0.00 |
|  | Spearman *r* | 0.38 | 0.10 | 0.09 | 0.37 | 0.05 | 0.04 | 0.17 | -0.14 | -0.10 | 0.07 | -0.14 | 0.04 | -0.20 | 0.03 |

Pearson and spearman coefficients extracted from GenomScape and Xena browsers. Gray shading indicates the selected threshold for significance obtained. * In cBioportal analysis of TCGA data, significance was not present (*IRF1* and *PD-L1*: Pearson *r* = 0.30, Spearman *r* = 0.36; *JAK2* and *PD-L2*: Pearson *r* = 0.28, Spearman *r* = 0.70).
