# Supplementary material for: PD-L1 and PD-L2 expression correlated genes in non-small-cell lung cancer
Source: Cancer Commun (Lond). 2019 Jun 3;39:30. doi: 10.1186/s40880-019-0376-6 (PMC6545701; doi:10.1186/s40880-019-0376-6)
Supplement: Supplementary file 6 — Additional file 6: Table S5. GSEA of IRF1 and IRF9 expression correlated genes in CCLE dataset (Lung_NSC) and TCGA datasets (LUAD and LUSC). [file 40880_2019_376_MOESM6_ESM.docx]

**Table S5** GSEA of *IRF1* and *IRF9* expression correlated genes in CCLE dataset (Lung_NSC) and TCGA dataset (LUAD and LUSC).

| **MSigDB hallmark gene set** | **K** | **Description of gene set** | **k** | **k/K** | ***P*** | ***q*** |
| --- | --- | --- | --- | --- | --- | --- |
| ***IRF1* expression correlated genes** | | | | | | |
| **CCLE dataset (Lung_NSC) (n = 152)** | | | | | | |
| Interferon gamma response | 200 | Genes up-regulated in response to IFNG [GeneID=3458]. | 38 | 0.19 | 3.83E-57 | 1.91E-55 |
| Interferon alpha response | 97 | Genes up-regulated in response to alpha interferon proteins. | 29 | 0.30 | 5.51E-50 | 1.38E-48 |
| TNFa signaling via NFKB | 200 | Genes regulated by NF-kB in response to TNF [GeneID=7124]. | 17 | 0.09 | 1.30E-19 | 2.17E-18 |
| Allograft rejection | 200 | Genes up-regulated during transplant rejection. | 11 | 0.06 | 4.90E-11 | 6.12E-10 |
| Inflammatory response | 200 | Genes defining inflammatory response. | 9 | 0.05 | 1.70E-08 | 1.70E-07 |
| IL6, JAK, STAT3 signaling | 87 | Genes up-regulated by IL6 [GeneID=3569] via STAT3 [GeneID=6774], e.g., during acute phase response. | 6 | 0.07 | 3.64E-07 | 3.03E-06 |
| Hypoxia | 200 | Genes up-regulated in response to low oxygen levels (hypoxia). | 7 | 0.04 | 3.70E-06 | 2.64E-05 |
| Hedgehog signaling | 36 | Genes up-regulated by activation of hedgehog signaling. | 4 | 0.11 | 5.18E-06 | 3.24E-05 |
| TGFb signaling | 54 | Genes up-regulated in response to TGFB1 [GeneID=7040]. | 4 | 0.07 | 2.66E-05 | 1.48E-04 |
| Complement | 200 | Genes encoding components of the complement system, which is part of the innate immune system. | 6 | 0.03 | 4.43E-05 | 2.02E-04 |
| **TCGA dataset (LUAD) (n = 559)** | | | | | | |
| Allograft rejection | 200 | Genes up-regulated during transplant rejection. | 85 | 0.43 | 2.12E-111 | 1.06E-109 |
| Interferon gamma response | 200 | Genes up-regulated in response to IFNG [GeneID=3458]. | 81 | 0.41 | 5.96E-104 | 1.49E-102 |
| Interferon alpha response | 97 | Genes up-regulated in response to alpha interferon proteins. | 43 | 0.44 | 2.11E-57 | 3.51E-56 |
| Inflammatory response | 200 | Genes defining inflammatory response. | 41 | 0.21 | 7.41E-39 | 9.26E-38 |
| Complement | 200 | Genes encoding components of the complement system, which is part of the innate immune system. | 32 | 0.16 | 5.57E-27 | 5.57E-26 |
| KRAS signaling up | 200 | Genes up-regulated by KRAS activation. | 27 | 0.14 | 5.91E-21 | 4.22E-20 |
| TNFA signaling via NFKB | 200 | Genes regulated by NF-kB in response to TNF [GeneID=7124]. | 27 | 0.14 | 5.91E-21 | 4.22E-20 |
| IL6, JAK, STAT3 Signaling | 87 | Genes up-regulated by IL6 [GeneID=3569] via STAT3 [GeneID=6774], e.g., during acute phase response. | 20 | 0.23 | 1.17E-20 | 7.32E-20 |
| IL2 STAT5 signaling | 200 | Genes up-regulated by STAT5 in response to IL2 stimulation. | 23 | 0.12 | 1.78E-16 | 9.89E-16 |
| Apoptosis | 161 | Genes mediating programmed cell death (apoptosis) by activation of caspases. | 11 | 0.07 | 2.78E-06 | 1.39E-05 |
| PI3K, AKT, MTOR signaling | 105 | Genes up-regulated by activation of the PI3K/AKT/mTOR pathway. | 7 | 0.07 | 2.12E-04 | 9.62E-04 |
| **TCGA dataset (LUSC) (n = 751)** | | | | | | |
| Interferon gamma response | 200 | Genes up-regulated in response to IFNG [GeneID=3458]. | 111 | 0.56 | 2.80E-147 | 1.40E-145 |
| Allograft rejection | 200 | Genes up-regulated during transplant rejection. | 98 | 0.49 | 1.73E-122 | 4.32E-121 |
| Interferon alpha response | 97 | Genes up-regulated in response to alpha interferon proteins. | 58 | 0.60 | 1.64E-79 | 2.74E-78 |
| Inflammatory response | 200 | Genes defining inflammatory response. | 55 | 0.28 | 4.68E-52 | 5.85E-51 |
| Complement | 200 | Genes encoding components of the complement system, which is part of the innate immune system. | 43 | 0.22 | 6.19E-36 | 6.19E-35 |
| TNFA signaling via NFKB | 200 | Genes regulated by NF-kB in response to TNF [GeneID=7124]. | 37 | 0.19 | 1.41E-28 | 1.17E-27 |
| IL2 STAT5 signaling | 200 | Genes up-regulated by STAT5 in response to IL2 stimulation. | 35 | 0.18 | 3.06E-26 | 2.19E-25 |
| KRAS signaling up | 200 | Genes up-regulated by KRAS activation. | 34 | 0.17 | 4.27E-25 | 2.67E-24 |
| IL6, JAK, STAT3 Signaling | 87 | Genes up-regulated by IL6 [GeneID=3569] via STAT3 [GeneID=6774], e.g., during acute phase response. | 25 | 0.29 | 7.52E-25 | 4.18E-24 |
| Apoptosis | 161 | Genes mediating programmed cell death (apoptosis) by activation of caspases. | 19 | 0.12 | 1.06E-11 | 5.31E-11 |
| ***IRF9* expression correlated genes** | | | | | | |
| **CCLE dataset (Lung_NSC) (n = 248)** | | | | | | |
| Interferon gamma response | 200 | Genes up-regulated in response to IFNG [GeneID=3458]. | 50 | 0.25 | 4.05E-70 | 2.03E-68 |
| Interferon alpha response | 97 | Genes up-regulated in response to alpha interferon proteins. | 39 | 0.40 | 3.54E-64 | 8.85E-63 |
| TNFA signaling via NFKB | 200 | Genes regulated by NF-kB in response to TNF [GeneID=7124]. | 16 | 0.08 | 1.10E-14 | 1.83E-13 |
| IL6, JAK, STAT3 Signaling | 87 | Genes up-regulated by IL6 [GeneID=3569] via STAT3 [GeneID=6774], e.g., during acute phase response. | 10 | 0.11 | 3.11E-11 | 3.89E-10 |
| Inflammatory response | 200 | Genes defining inflammatory response. | 13 | 0.07 | 4.95E-11 | 4.95E-10 |
| Hypoxia | 200 | Genes up-regulated in response to low oxygen levels (hypoxia). | 10 | 0.05 | 1.05E-07 | 8.75E-07 |
| Complement | 200 | Genes encoding components of the complement system, which is part of the innate immune system. | 9 | 0.05 | 1.11E-06 | 6.16E-06 |
| KRAS signaling up | 200 | Genes up-regulated by KRAS activation. | 9 | 0.05 | 1.11E-06 | 6.16E-06 |
| MTORC1 signaling | 200 | Genes up-regulated through activation of mTORC1 complex. | 9 | 0.05 | 1.11E-06 | 6.16E-06 |
| Allograft rejection | 200 | Genes up-regulated during transplant rejection. | 8 | 0.04 | 1.05E-05 | 4.76E-05 |
| **TCGA dataset (LUAD) (n = 82)** | | | | | | |
| Interferon gamma response | 200 | Genes up-regulated in response to IFNG [GeneID=3458]. | 52 | 0.26 | 3.06E-105 | 1.53E-103 |
| Interferon alpha response | 97 | Genes up-regulated in response to alpha interferon proteins. | 45 | 0.46 | 1.06E-103 | 2.66E-102 |
| Allograft rejection | 200 | Genes up-regulated during transplant rejection. | 8 | 0.04 | 2.48E-09 | 4.13E-08 |
| Complement | 200 | Genes encoding components of the complement system, which is part of the innate immune system. | 7 | 0.04 | 6.46E-08 | 6.46E-07 |
| Inflammatory response | 200 | Genes defining inflammatory response. | 7 | 0.04 | 6.46E-08 | 6.46E-07 |
| TNFA signaling via NFKB | 200 | Genes regulated by NF-kB in response to TNF [GeneID=7124]. | 5 | 0.03 | 2.74E-05 | 2.28E-04 |
| **TCGA dataset (LUSC) (n = 90)** | | | | | | |
| Interferon gamma response | 200 | Genes up-regulated in response to IFNG [GeneID=3458]. | 57 | 0.29 | 1.89E-115 | 9.46E-114 |
| Interferon alpha response | 97 | Genes up-regulated in response to alpha interferon proteins. | 47 | 0.48 | 4.92E-107 | 1.23E-105 |
| Allograft rejection | 200 | Genes up-regulated during transplant rejection. | 10 | 0.05 | 6.53E-12 | 1.09E-10 |
| Inflammatory response | 200 | Genes defining inflammatory response. | 8 | 0.04 | 5.33E-09 | 6.67E-08 |
| IL6, JAK, STAT3 Signaling | 87 | Genes up-regulated by IL6 [GeneID=3569] via STAT3 [GeneID=6774], e.g., during acute phase response. | 6 | 0.07 | 1.85E-08 | 1.85E-07 |
| TNFa signaling via NFKB | 200 | Genes regulated by NF-kB in response to TNF [GeneID=7124]. | 7 | 0.04 | 1.25E-07 | 1.04E-06 |
| Apoptosis | 161 | Genes mediating programmed cell death (apoptosis) by activation of caspases. | 5 | 0.03 | 1.54E-05 | 1.10E-04 |

K, number of genes in gene-set; k, number of genes in overlap. NS, no significant associations**.** *P*, P value; *q*, q value for FDR. Cut off FDR q value < 1E-03.
