# Supplementary material for: PD-L1 and PD-L2 expression correlated genes in non-small-cell lung cancer
Source: Cancer Commun (Lond). 2019 Jun 3;39:30. doi: 10.1186/s40880-019-0376-6 (PMC6545701; doi:10.1186/s40880-019-0376-6)
Supplement: Supplementary file 7 — Additional file 7: Table S6. Differences in expression in TCGA datasets (LUAD and LUSC) versus normal paired tissue from GTEx and TCGA. [file 40880_2019_376_MOESM7_ESM.docx]

**Table S6** Differences in expression in TCGA dataset (LUAD and LUSC) versus normal paired tissue from GTEx and TCGA.

| **Gene** | **TCGA dataset** | **TPM** | **Fold change**  **tumor/normal (dataset GTEx)** | **Fold change**  **tumor/normal (dataset TCGA)** | **Wilcoxon *P* value**  **tumor/normal (dataset TCGA)** |
| --- | --- | --- | --- | --- | --- |
| *PD-L1* | LUAD | 4 | 0.3 | 0.4 | 6.9E-13 |
|  | LUSC | 5 | 0.3 | 0.5 | 1.3E-7 |
| *PD-L2* | LUAD | 2 | 0.5 | 0.3 | 1.2E-17 |
|  | LUSC | 2 | 0.5 | 0.4 | 6.3E-14 |
| *PD-1* | LUAD | 2 | 1.1 | 1.6 | 3.4E-6 |
|  | LUSC | 1 | 0.9 | 1.1 | 5.3E-1 |
| *CD80* | LUAD | 1 | 0.5 | 0.7 | 4.6E-7 |
|  | LUSC | 1 | 0.4 | 0.4 | 3.3E-15 |
| *RGMB* | LUAD | 6 | 0.7 | 0.7 | 1.9E-5 |
|  | LUSC | 6 | 0.7 | 0.8 | 1.5E-4 |

Fold-change calculated from RNA-sequencing transcripts per million (TPM) from http://gepia.cancer-pku.cn and <http://maplab.imppc.org/wanderer/>.

Fold change Wilcoxon *P* value calculated from <http://gepia.cancer-pku.cn> using tumor and normal paired tissue.
