# Supplementary material for: PD-L1 and PD-L2 expression correlated genes in non-small-cell lung cancer
Source: Cancer Commun (Lond). 2019 Jun 3;39:30. doi: 10.1186/s40880-019-0376-6 (PMC6545701; doi:10.1186/s40880-019-0376-6)
Supplement: Supplementary file 10 — Additional file 10: Table S8. Gene lists representing various subsets of PD-L1 and PD-L2 expression correlated genes in CCLE dataset (Lung_NSC) and TCGA datasets (LUAD and LUSC). [file 40880_2019_376_MOESM10_ESM.docx]

**Table S8** Gene lists representing various subsets of *PD-L1* and *PD-L2* expression correlated genes in CCLE dataset (Lung_NSC) and TCGA dataset (LUAD and LUSC).

| ***PD-L1*** | ***PD-L2*** | ***PD-L1*** | ***PD-L2*** | ***PD-L1*** | ***PD-L2*** | ***PD-L1*** | ***PD-L1*** |
| --- | --- | --- | --- | --- | --- | --- | --- |
| **Lung_NSC, LUAD and LUSC**  **(n = 4)** | **Lung_NSC, LUAD and LUSC**  **(n = 4)** | **Lung_NSC and LUSC**  **(n = 4)** | **Lung_NSC and LUSC**  **(n = 4)** | **Lung_NSC and LUAD**  **(n = 49)** | **Lung_NSC and LUAD**  **(n = 26)** | **LUSC and Chr9p24 localized**  **(n = 13)** | **LUSC and non-Chr9p24 localized**  **(n = 13)** |
| *PD-L1* | *PD-L1* | *PD-L1* | *PD-L1* | *PD-L1* | *PD-L1* | *PD-L1* | *GBP4* |
| *PD-L2* | *PD-L2* | *PD-L2* | *PD-L2* | *PD-L2* | *PD-L2* | *PD-L2* | *APOL6* |
| *APOL6* | *TRIM22* | *APOL6* | *TRIM22* | *STK10* | *STK10* | *SMARCA2* | *FASLG* |
| *PLGRKT* | *TMEM106A* | *PLGRKT* | *TMEM106A* | *RAC2* | *RAC2* | *CDC37L1* | *GPR174* |
|  |  |  |  | *PPP1R18* | *PPP1R18* | *AK3* | *SLA2* |
|  |  |  |  | *SAMD9L* | *SAMD9L* | *RCL1* | *STAT1* |
|  |  |  |  | *SAMD9* | *COTL1* | *JAK2* | *PRF1* |
|  |  |  |  | *PML* | *TRIM22* | *PLGRKT* | *TLR1* |
|  |  |  |  | *PARP12* | *MIR155HG* | *RIC1* | *TLR6* |
|  |  |  |  | *APOL6* | *HLA-E** | *RANBP6* | *GNA13* |
|  |  |  |  | *TAP2* | *ADAM19* | *KIAA2026* | *TDRD7* |
|  |  |  |  | *TNFRSF9* | *AXL* | *UHRF2** | *TTC39B* |
|  |  |  |  | *DTX3L* | *DSE* | *KDM4C* | *STX11* |
|  |  |  |  | *GBP1* | *ELK3* |  |  |
|  |  |  |  | *DAPP1* | *ETS1* |  |  |
|  |  |  |  | *DDX60* | *GPR176* |  |  |
|  |  |  |  | *AIM2* | *LAYN* |  |  |
|  |  |  |  | *TNFAIP3* | *LOX* |  |  |
|  |  |  |  | *IFIT3** | *PEA15* |  |  |
|  |  |  |  | *ARNTL2* | *PLAU* |  |  |
|  |  |  |  | *AXL* | *RIN3* |  |  |
|  |  |  |  | *BCL10* | *SPHK1* |  |  |
|  |  |  |  | *CCDC71L* | *SPIRE2* |  |  |
|  |  |  |  | *CD109* | *TUBB6* |  |  |
|  |  |  |  | *CDCP1* | *ZYX* |  |  |
|  |  |  |  | *CFLAR* | *TMEM106A* |  |  |
|  |  |  |  | *DCBLD2* |  |  |  |
|  |  |  |  | *DDX60L* |  |  |  |
|  |  |  |  | *EHD1* |  |  |  |
|  |  |  |  | *EHD4* |  |  |  |
|  |  |  |  | *ELK3* |  |  |  |
|  |  |  |  | *HIF1A* |  |  |  |
|  |  |  |  | *IDS* |  |  |  |
|  |  |  |  | *IFI16* |  |  |  |
|  |  |  |  | *MAN2A1* |  |  |  |
|  |  |  |  | *PIP4K2A* |  |  |  |
|  |  |  |  | *PLAU* |  |  |  |
|  |  |  |  | *PMAIP1* |  |  |  |
|  |  |  |  | *RALB* |  |  |  |
|  |  |  |  | *RGS10* |  |  |  |
|  |  |  |  | *ROR1* |  |  |  |
|  |  |  |  | *SLC9A7* |  |  |  |
|  |  |  |  | *SMURF2* |  |  |  |
|  |  |  |  | *SPATS2L* |  |  |  |
|  |  |  |  | *TGFBI* |  |  |  |
|  |  |  |  | *TMEM106A* |  |  |  |
|  |  |  |  | *TSC22D2* |  |  |  |
|  |  |  |  | *UBA6* |  |  |  |
|  |  |  |  | *PLGRKT* |  |  |  |

*excluded in downstream heatmap analyses according to lack of sufficient expression data.
