# Supplementary material for: PD-L1 and PD-L2 expression correlated genes in non-small-cell lung cancer
Source: Cancer Commun (Lond). 2019 Jun 3;39:30. doi: 10.1186/s40880-019-0376-6 (PMC6545701; doi:10.1186/s40880-019-0376-6)
Supplement: Supplementary file 12 — Additional file 12: Table S9. GSEA for genomic localization of PD-L1 and PD-L2 expression correlated genes. [file 40880_2019_376_MOESM12_ESM.docx]

**Table S9** GSEA for genomic localization of *PD-L1* and *PD-L2* expression correlated genes.

| **Gene** | **MSigDB chromosomal position** | **K** | **k** | **k/K** | ***P*** | ***q*** |
| --- | --- | --- | --- | --- | --- | --- |
| **CCLE dataset (Lung_NSC)** | | | | | | |
| *PD-L1* | chr12q12 | 73 | 8 | 0.11 | 8.21E-07 | 2.68E-04 |
| **TCGA dataset (LUAD)** | | | | | | |
| *PD-L1* | chr9p24 | 61 | 6 | 0.10 | 7.55E-07 | 2.46E-04 |
| *PD-L2* | chr12p13 | 270 | 28 | 0.10 | 2.94E-13 | 9.58E-11 |
| *PD-L2* | chr6p21 | 544 | 35 | 0.06 | 3.11E-10 | 5.07E-08 |
| *PD-L2* | chr19q13 | 948 | 45 | 0.05 | 1.49E-08 | 1.62E-06 |
| **TCGA dataset (LUSC)** | | | | | | |
| *PD-L1* | chr9p24 | 61 | 13 | 0.21 | 1.03E-31 | 3.36E-29 |
| *PD-L2* | chr19q13 | 948 | 23 | 0.02 | 2.62E-07 | 6.58E-05 |
| *PD-L2* | chr12p13 | 270 | 12 | 0.04 | 4.89E-07 | 6.58E-05 |
| *PD-L2* | chr1q23 | 139 | 9 | 0.06 | 6.06E-07 | 6.58E-05 |
| *PD-L2* | chr9p24 | 61 | 6 | 0.10 | 4.22E-06 | 3.44E-04 |

K, number of genes in gene-set; k, number of genes in overlap; *P*, *P* value; q, *q* value for FDR. Cut off FDR q value < 1E-03.
